# Uncertainty in vulnerability of networks under attack

**DOI:** 10.1038/s41598-023-29899-w

**Published:** 2023-02-23

**Authors:** Alireza Ermagun, Nazanin Tajik, Hani Mahmassani

**Affiliations:** 1 Department of Geography and Geoinformation Science, George Mason University, 4400 University Dr., Fairfax, VA 22030 USA; 2 Department of Industrial and Systems Engineering, Mississippi State University, 501 Hardy Road, 260 McCain Hall, Mississippi State, MS 39762 USA; 3 Department of Civil and Environmental Engineering, Northwestern University, 2145 Sheridan Road, Evanston, IL 60208 USA

**Keywords:** Engineering, Complex networks

## Abstract

This study builds conceptual explanations and empirical examinations of the vulnerability response of networks under attack. Two quantities of “vulnerability” and “uncertainty in vulnerability” are defined by scrutinizing the performance loss trajectory of networks experiencing attacks. Both vulnerability and uncertainty in vulnerability quantities are a function of the network topology and size. This is tested on 16 distinct topologies appearing in infrastructure, social, and biological networks with 8 to 26 nodes under two percolation scenarios exemplifying benign and malicious attacks. The findings imply (i) crossing path, tree, and diverging tail are the most vulnerable topologies, (ii) complete and matching pairs are the least vulnerable topologies, (iii) complete grid and complete topologies show the most uncertainty for vulnerability, and (iv) hub-and-spoke and double u exhibit the least uncertainty in vulnerability. The findings also imply that both vulnerability and uncertainty in vulnerability increase with an increase in the size of the network. It is argued that in networks with no undirected cycle and one undirected cycle, the uncertainty in vulnerability is maximal earlier in the percolation process. With an increase in the number of cycles, the uncertainty in vulnerability is accumulated at the end of the percolation process. This emphasizes the role of tailoring preparedness, response, and recovery phases for networks with different topologies when they might experience disruption.

## Introduction

From the six handshakes rule^1,2^ and the random graph theory^3^ to the theory of evolving networks^4^, much research in network science is grappling with comprehending the nexus of network structure and function. This, in part, is due to the need to understand the overall system’s response under peculiar circumstances. Malicious and random attacks induced by nature or humans are pertinent examples^5–7^. As our daily lives depend on the functioning of networks ranging from infrastructure (e.g., power grids, transport, gas, water) to social (e.g., communication, social media) and biological (e.g., neural, metabolic), research has proliferated to examine the vulnerability of networks under percolation^5–16^.

Vulnerability is critical when pondering on the ability of a network to provide continuity in its operation. It refers to the magnitude of network performance loss relative to its pre-disruption performance in the presence of disruptions and the absence of response resources^17^. Recent events including the spread of COVID-19 through individuals over the networks of contacts in 2020^18^, the shutdown of the New York City subway system following Hurricane Ida in 2021^19^, and the power outage experienced by 2 million Texan households induced by a massive winter storm in 2021^20^ have shown how the vulnerability response of systems exacerbates the effect of attacks on our daily lives, with severe social and economic consequences.

Much of the previous research has contributed to examining the vulnerability of networks through the lens of percolation in which links are removed or become non-functional uniformly at random or in order of their degrees^10–12,21–25^. It includes sequential single link removal^16,26^, sequential multiple link removal^6,7^, randomly chosen multiple link removal^5,6^, and targeted multiple link removal^5,6^. Examples of examining the vulnerability of networks under random, targeted, and cascading disruptions^5,6,15^ exist in traffic networks (e.g., Sioux Falls)^6,16^, public transit networks (e.g., bus and subway network in Beijing, China)^7^, power grid networks (e.g., Continental European Transmission Network)^5^, and complex networks (e.g., Erdős-Rényi and Barabási-Albert scale free networks)^15^. Little is known about the vulnerability response of networks with different topologies and sizes under different attack scenarios. This study is a natural continuation of previous research and contributes to understanding the vulnerability response of networks by (i) introducing the concept of “uncertainty in vulnerability” and (ii) visualizing and measuring the vulnerability of networks with different topologies and sizes when links are removed in order of their degrees. This happens by starting with the highest degrees and working down, then using the lowest degrees and working up while recalculating the residual network flow in each iteration.

Here, our overarching goal is to equip scientists and practitioners with the knowledge and tools necessary to understand the vulnerability response of networks in relation to the network topology. We define two quantities to characterize the vulnerability response. The first quantity, $V\left(G\left(N,L\right)\right)$, is the vulnerability of a network under benign and malicious attacks and indicates how performance loss happens when links are removed by percolation. The second quantity, $U\left(G\left(N,L\right)\right)$, is the uncertainty in vulnerability and simply measures the area between the upper and lower bounds of performance loss trajectories. It implies the threshold of uncertainty in the network performance degradation. Both vulnerability and uncertainty in vulnerability quantities are a function of the network topology and size. This is tested on 16 distinct topologies appearing in infrastructure, social, and biological networks with 8 to 26 nodes under two percolation scenarios exemplifying benign and malicious attacks. Vulnerability and uncertainty in vulnerability quantities are established next, results and discussion follow, and materials and methods are described thereafter.

## Vulnerability and uncertainty in vulnerability

**Figure 1:**
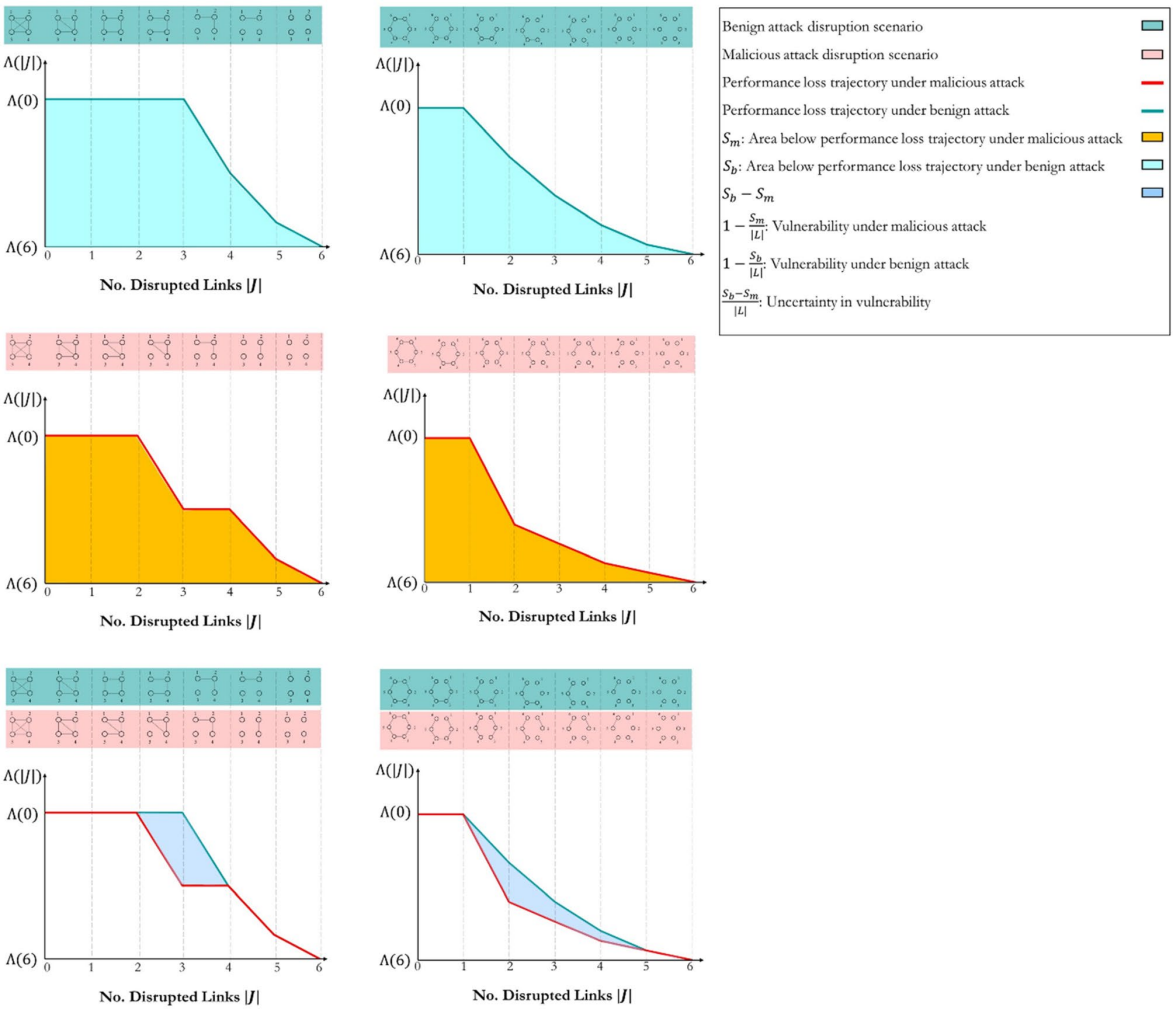
Performance loss trajectory of the ring and complete networks with six links. The figure represents the interdependencies among the network topology, redundancy level, and the delay in the network performance loss trajectory. Considering complete network topology, the disruption of three links $(\left|J\right|=3)$ does not change the performance of the networks from their original performance and ${\varphi }_{\left|L\right|-|J| }={\varphi }_{|L|}$. We draw a similar conclusion for ring network topology with the disruption of one link.

We define network $G=\left(N,L\right)$ as an undirected graph with $|N|$ number of nodes connected by $|L|$ number of links. Let us assume ${\varphi }_{\left|L\right|}$ as the network’s performance at the original state showing the total delivered demand throughout the network. Employing the bond percolation, we remove links in order of their betweenness centrality under benign and malicious attack scenarios as depicted in Fig. 1. In the benign attack scenario, links are removed from lowest to highest, while links are removed from highest to lowest in the malicious attack scenario. The betweenness centrality of links is recalculated following each removal in both scenarios as the network topology and betweenness centrality of links does not necessarily remain identical. Departing from the original state, ${\varphi }_{\left|L\right|-|J|}$ becomes the network performance after the elimination of $\left|J\right|$ number of links, where $J\subseteq L$ is the set of disrupted links. Mathematically, we explain the general form of network flow models to calculate the network performance ${\mathrm{\varphi }}_{\left|\mathrm{L}\right|-|\mathrm{J}|}$ in Materials and Methods.

Following the disruption of ${i}^{th}$ link, two regular conditions emerge:

- ${{\varphi }_{\left|L\right|-i}=\varphi }_{\left|L\right|}$: The network’s performance is unaffected by the attack if the total delivered demand through the network equals the total delivered demand at the original state.
- ${{\varphi }_{\left|L\right|-i}<\varphi }_{\left|L\right|}$: The network’s performance deteriorates by the attack, and the total delivered demand through the network is less than the total delivered demand at the original state. A singular condition is ${\varphi }_{\left|L\right|-i}=0$, when the total delivered demand equals zero, and the network is completely disrupted.

A peculiar condition is when ${{\varphi }_{\left|L\right|-i}>\varphi }_{\left|L\right|}$ and it might happen when the network’s performance is improved by the attack. A pertinent example is Braess’s paradox^27^. Here, we do not exercise this condition as we assume a uniform fixed link cost and uniform demand. This assumption, however, is not restrictive as our algorithm generates two non-inferior trajectories that postulate the performance bounds under benign and malicious attacks. These proposed bounds encompass the performance loss trajectory of network topologies with different link characteristics.

Equation (1) mathematically examines the relative performance of the network, where $\Lambda \left(i\right)$ ranges from 0 to 1.

1 $$\Lambda \left(i\right)=\frac{{\varphi }_{\left|L\right|-i}}{{\varphi }_{\left|L\right|}}$$

When $\Lambda \left(i\right)=1$, the network consists of redundant links, and their removal does not lead to performance loss. Equation (2) measures the redundancy of the network, where $\left|L\right|$ is the cardinality of the set of operational links at the original state, $\left|J\right|$ is the cardinality of the set of disrupted links, and $H$ is any possible set of disrupted links in network $G$. Simply, $|J|$ shows the maximum number of links that can be disrupted within network $G$ without deteriorating the original state performance, and $J\subseteq L$.

2 $$\mathcal{R}\mathfrak{e}\left(G\left(N,L\right)\right)=1-\frac{\left|J\right|}{\left|L\right|} , J\subseteq L \mathrm{and} J=\underset{{\varphi }_{\left|L\right|-|H|}={\varphi }_{\left|L\right|}}{\mathrm{max}}|H|$$

The following considerations are important:

- The maximum value of $\mathcal{R}\mathfrak{e}\left(G\left(N,L\right)\right)$ equals one. This is obtained when $\left|J\right|=0$ or the network has no redundancy.
- The minimum value of $\mathcal{R}\mathfrak{e}\left(G\left(N,L\right)\right)$ equals $\lim_{\left| N \right| \to + \infty } \left( {1 - \frac{\left| N \right| - 2}{{\left| N \right|}}} \right) \approx 0$. This is obtained when network $G$ is a complete network with a considerably large set of nodes or a high redundancy level.

Following the relative performance and redundancy definition, we formally define vulnerability under benign and malicious attacks in Eqs. (3) and (4), where ${\Lambda }_{\mathrm{b}}$ is the relative performance in the presence of the benign attack and ${\Lambda }_{\mathrm{m}}$ is the relative performance in the presence of the malicious attack in network $G$ with $|L|$ number of links. ${S}_{b}$ is the area below network performance loss trajectory under benign attack and ${S}_{m}$ is the area below network performance loss trajectory under malicious attack.

3 $$\begin{gathered} V_{b} \left( {G\left( {N,L} \right)} \right) = 1 - \frac{{S_{b} }}{\left| L \right|} \hfill \\ S_{b} = \mathop \sum \limits_{i = 0}^{\left| L \right|} \frac{{\left( {{\Lambda }_{{\text{b}}} \left( i \right) + {\Lambda }_{{\text{b}}} \left( {i + 1} \right)} \right)}}{2} \hfill \\ \end{gathered}$$

4 $$\begin{gathered} V_{m} \left( {G\left( {N,L} \right)} \right) = 1 - \frac{{S_{m} }}{\left| L \right|} \hfill \\ S_{m} = \mathop \sum \limits_{i = 0}^{\left| L \right|} \frac{{\left( {{\Lambda }_{{\text{m}}} \left( i \right) + {\Lambda }_{{\text{m}}} \left( {i + 1} \right)} \right)}}{2} \hfill \\ \end{gathered}$$

Immediately, the uncertainty in vulnerability can be introduced by Eq. (5). This is the link-normalized area between performance loss trajectories under benign and malicious attacks. It represents the uncertainty in vulnerability response when the network experiences random attacks falling between two extreme attack boundaries. This quantity is always non-negative as ${S}_{b}>{S}_{m}$ and ${V}_{m}\left(G\left(N,L\right)\right)>{V}_{b}\left(G\left(N,L\right)\right)$. As ${\Lambda }_{m}\left(i\right)\le {\Lambda }\left(i\right)\le {\Lambda }_{b}\left(i\right)$, it can simply be proved that ${V}_{m}\left(G\left(N,L\right)\right)\le V\left(G\left(N,L\right)\right)\le {V}_{b}\left(G\left(N,L\right)\right)$.

5 $$U\left( {G\left( {N,L} \right)} \right) = \frac{{S_{b} - S_{m} }}{\left| L \right|} = V_{m} \left( {G\left( {N,L} \right)} \right) - V_{b} \left( {G\left( {N,L} \right)} \right)$$

## Results

### Vulnerability under attacks

**Figure 2:**
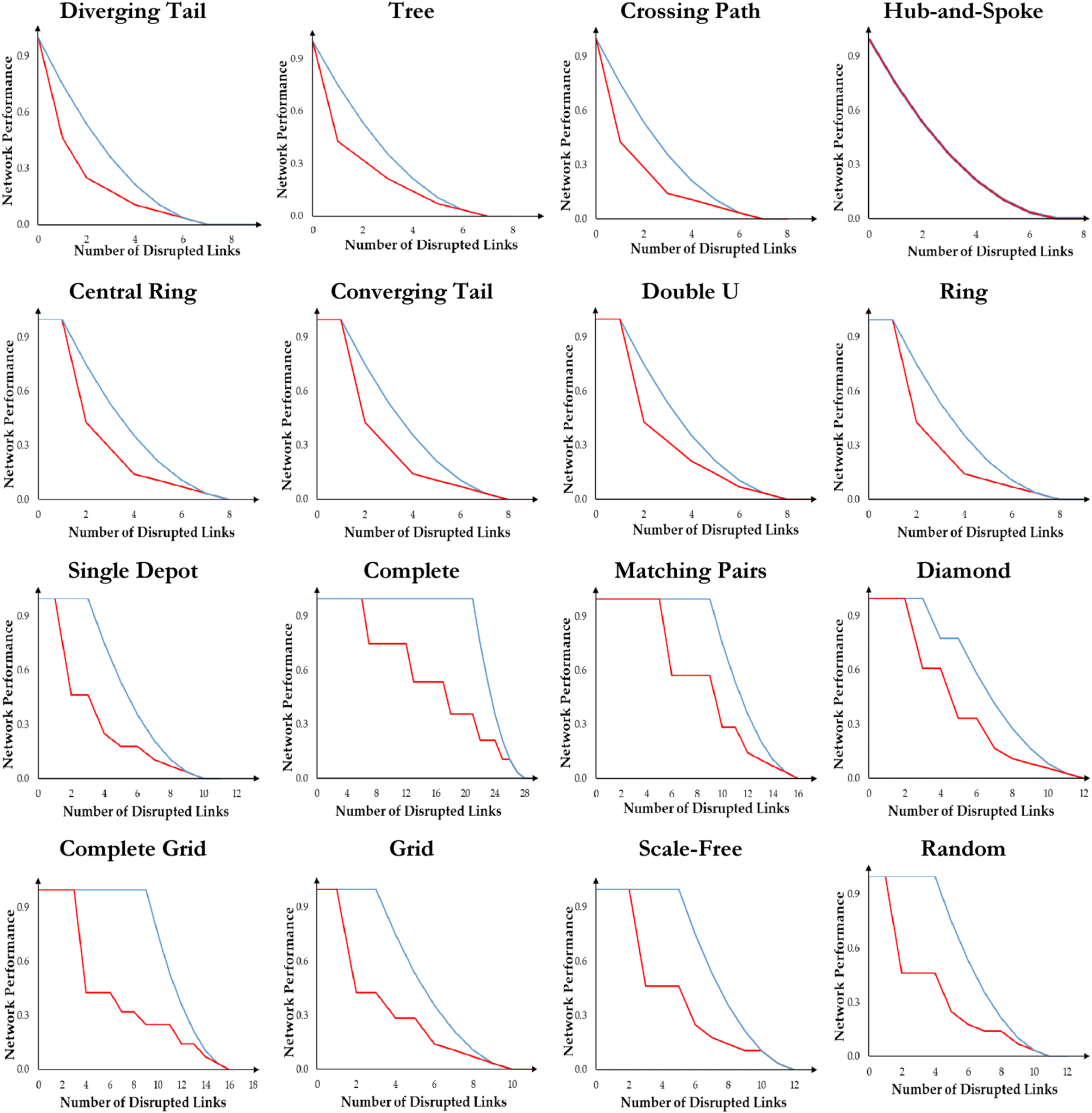
Disruption trajectory of 16 network topologies with 8 nodes. The x-axis represents the number of removed links. The y-axis represents the normalized network performance. Three observations are noted. First, topologies with no redundancy, such as Diverging Tail, Tree, Crossing Path, and Hub-and-Spoke, follow similar disruption trajectories with no delay in network performance devolution. For these topologies $\mathcal{R}\mathfrak{e}\left(G\left(N,L\right)\right)$ equals 1. Second, topologies with one undirected cycle, such as Central Ring, Converging Tail, Ring, and Double U, have one unit of delay in network performance degradations. After disconnecting the cycle, they follow disruption trajectories like the topologies with no redundancy. The redundancy measure for such networks equals $\frac{\left|N\right|-1}{|N|}$. Third, in the presence of a malicious attack, the increase in the minimum number of undirected cycles in the network increases delays in the network performance degradations at the beginning of the disruption process. The minimum number of cycles in a network equals the minimum existing node degree in the network minus one. The number and length of delays in the middle of the disruptions process depends, respectively, on the existence of nodes with a minimum degree greater than two in the residual network.

We visualize the performance loss trajectory of network disruptions for 16 topologies with different sizes in physical, social, meteorological, and biological systems documented in Supplementary Information I. We depict the disruption trajectory from the original state to the complete disruption when the network experiences an attack for each topology. Many attack scenarios are possible. However, all attack scenarios fall in a range limited by two extreme scenarios of malicious and benign forming the lower and upper boundaries as formally established in Supplementary Information II*.* The malicious and benign attack scenarios are respectively linked to the maximum and minimum performance loss rate in each link removal step. We employ both attack scenarios on a range of network sizes from 8 to 26 nodes. Although this range is noted in existing analysis of actual networks with examples of transport networks^28,29^, gas networks^30^, power networks^31^, and water networks^32^, it is selected to exemplify how vulnerability and uncertainty in vulnerability is a function of the size of the network. We document the generalization of our findings for 16 network topologies with more than 26 nodes in Supplementary Information II. The propositions posed in Supplementary Information II utilize mathematical induction and proof by contradiction to estimate the bounds of network performance deterioration when network size goes to infinity. This assures the consistency of our conclusions regardless of the network size. Figure 2 visualizes the trajectory of network disruptions with 8 nodes. A complete set of trajectories are documented in Supplementary Information III.

**Figure 3:**
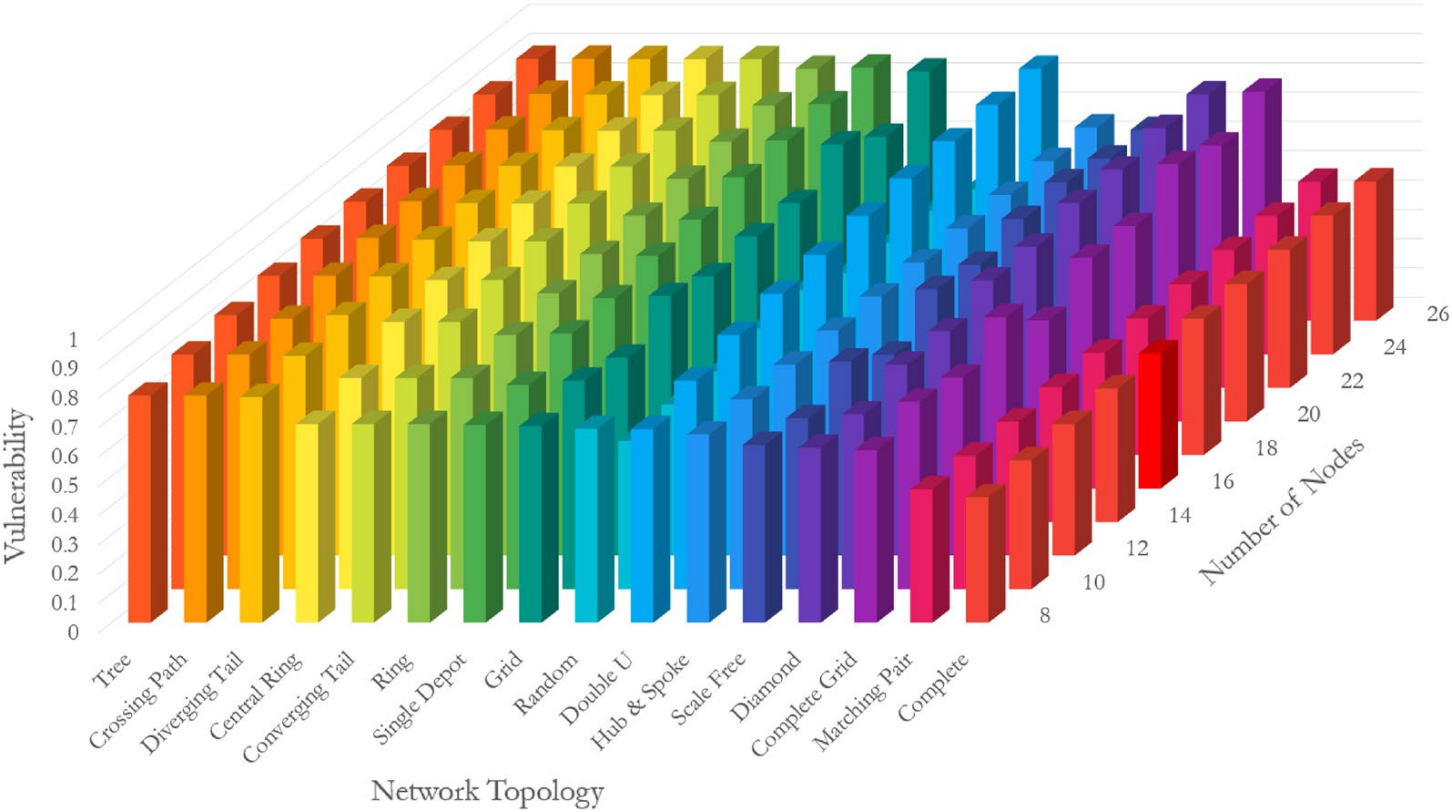
Vulnerability of networks with different topologies and sizes.

Figure 3 depicts the vulnerability of sixteen network topologies with 8–26 nodes. Three observations are noted. First, complete and matching pair topologies have almost the least vulnerability regardless of the network size. Their vulnerability quantity ranges between 0.43 and 0.47 depending on the size of the network. The opposites are tree, crossing path, and diverging tail topologies with the most vulnerability. Their vulnerability quantity ranges between 0.77 and 0.90 depending on the size of the network. It means the difference between the topology of networks can result in up to 2 times more vulnerable systems, accentuating the role of topology in the vulnerability of networks. Second, the network experiences an increase in vulnerability with an increase in the size of the network, although the rate of escalation is a function of the topology. The vulnerability of complete grid, central ring, converging tail, and double u increases with the highest rate of 32.5%, 31.5%, 31.5%, and 30.0%, respectively when the number of nodes increases from 8 to 26. Hub-and-spoke and matching pair, quite the opposite, manifest the lowest rate of change in their vulnerability with an increase in the size of the network. The vulnerability of hub-and-spoke and matching pair topologies increases with a rate of 2.6% and 4.0% when the number of nodes enlarges from 8 to 26. Third, by nature, the vulnerability of the random topology responds differently to the size of the network as its rewiring follows a random probability distribution. The random topology consistently dwells amongst low vulnerable topologies, particularly when its rewiring probability is greater than 0.5. Propositions 1–5 in Supplementary Information II adopt these observations to prove the applicability of our findings by mathematical induction for networks of sizes greater than 26 nodes.

**Figure 4:**
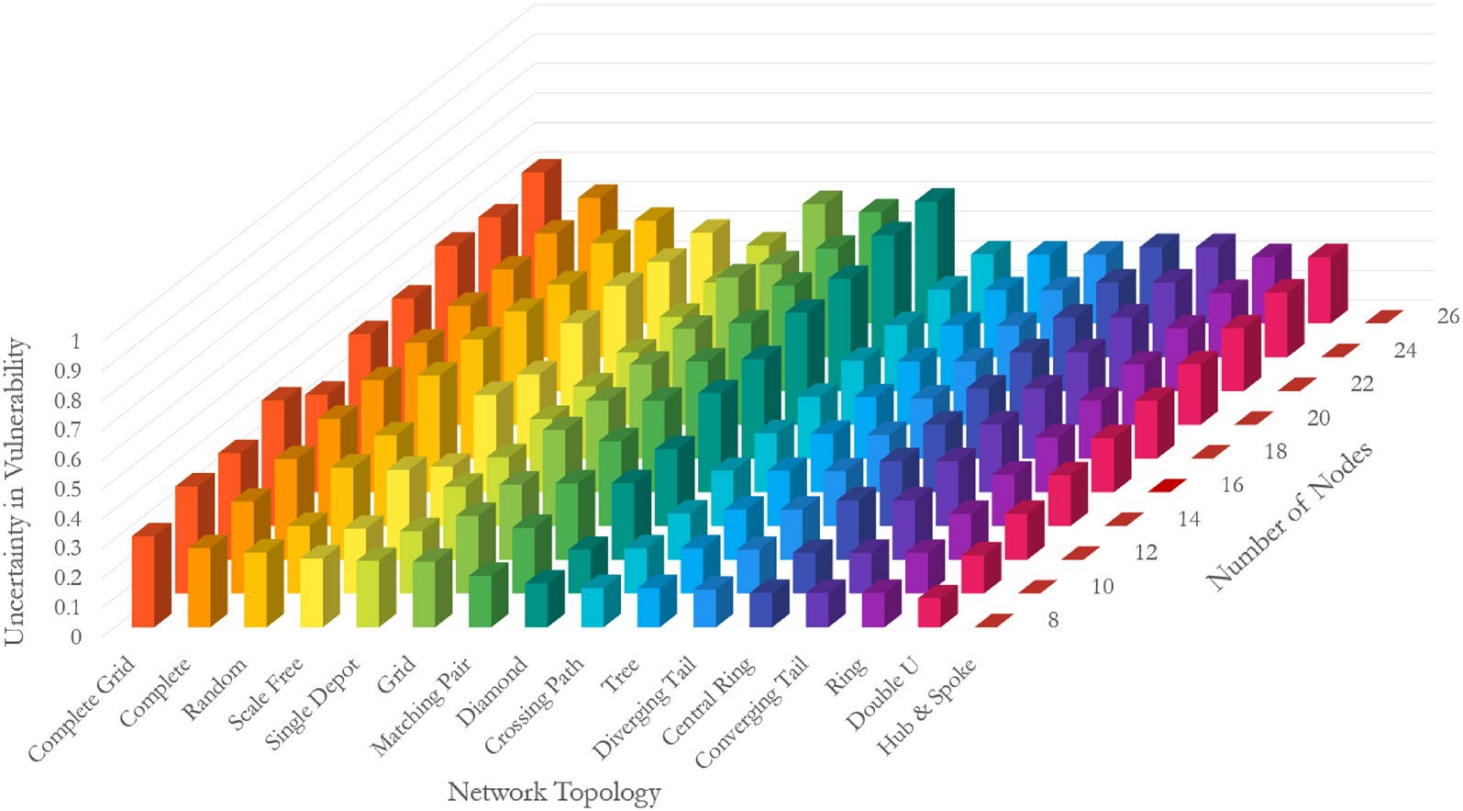
Uncertainty in the vulnerability of networks with different topologies and sizes.

A similar examination on uncertainty in vulnerability leads to conclusive findings derived from Fig. 4. First, hub-and-spoke, double u, and ring topologies have the lowest uncertainty in vulnerability, regardless of the size of the network. Their uncertainty in vulnerability quantity ranges between 0 and 0.22 depending on the size of the network. On the contrary, the uncertainty in vulnerability of complete grid topologies ranges from 0.26 to 0.51, depending on the size of the network. Ignoring the hub-and-spoke topology with no uncertainty in vulnerability, the difference between the topology of networks can result in more than 2 times more uncertain systems. Second, like the pattern detected in vulnerability trajectories, the network experiences an increase in the uncertainty in vulnerability with an increase in the size of the network. However, the rate of escalation is a function of the topology. The uncertainty in the vulnerability of diamond and double u increases with the highest rate of 178.4% and 128.8%, respectively, when the number of nodes increases from 8 to 26. Hub-and-spoke and single depot topologies manifest the lowest rate of change in their uncertainty in vulnerability with an increased rate of 0% and 17.4% when the number of nodes enlarges from 8 to 26. A comparison between vulnerability and uncertainty in vulnerability reveals that (i) the increase rate in uncertainty in vulnerability is significantly more than the increase rate in vulnerability when the network size increase and it is not topology-dependent, (ii) the hub-and-spoke topology experiences the least increase in vulnerability and no changes in uncertainty in vulnerability with an increase in the size of the network, and (iii) the complete topology exhibits the most vulnerability and uncertainty in vulnerability. Our conclusions are drawn under the link percolation assumption, while the node percolation can be more debilitating in certain instances. For example, the hub-and-spoke topology is extremely vulnerable to an attack on the hub node. Proposition 6 in Supplementary Information II adopts these examinations and uses proof by induction to generalize our findings for networks of sizes greater than 26 nodes.

### Uncertainty in vulnerability distribution

We argue that uncertainty in vulnerability is not a uniform area and is a function of the topology, as observed in Fig. 2, and the size of the network. This happens due to an unequal difference between performance loss under benign and malicious attacks following each link removal. An illustrative example suffices. Looking at the performance loss trajectory of the complete topology depicted in Fig. 2, it is noticed that both trajectories overlap earlier in the percolation process. The uncertainty in vulnerability, therefore, equals zero. Moving forward in the percolation process, a sudden gap is created, showing the difference between the performance loss when the complete network is under benign and malicious attacks. The gap widens but eventually narrows as more links are removed from the network. Mathematically, it is calculated by Eq. (6). Here, ${\Lambda }_{b}\left(i\right)$ is the network performance after the ${i}^{th}$ link is disrupted under the benign attack, and ${\Lambda }_{m}\left(i\right)$ is the network performance after the ${i}^{th}$ link is disrupted under the malicious attack.

6 $$\mathcal{R}\left(i\right)= {\Lambda }_{b}\left(i\right)-{\Lambda }_{m}\left(i\right) i=1,\dots ,\left|L\right|$$

**Figure 5:**
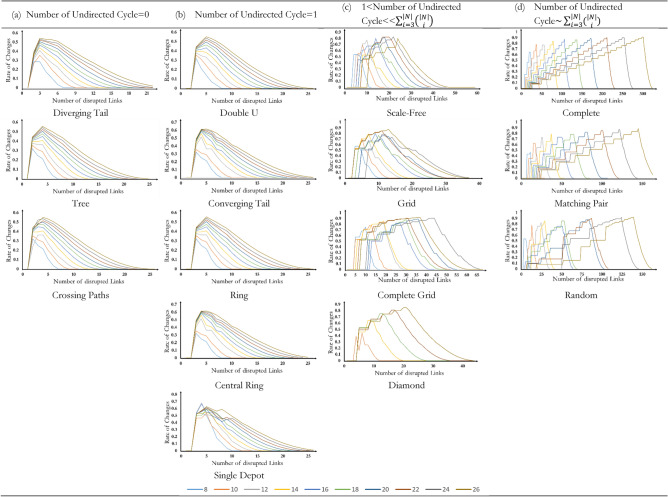
Trajectory of R(.) for 16 network topologies with 8 to 26 nodes but hub-and-spoke as its performance loss trajectory under benign attack is the same as its performance loss trajectory under malicious attack. (**a**) Regardless of topological characteristics, the rate of change in networks with no cycle follows a polynomial trajectory with a degree of four. (**b**) The existence of one cycle would not change the regression function but shift the trendline one unit forward. (**c**) The network topologies follow polynomial functions with the degree of five. As the number of cycles increases, the skewness of the regression function decreases. Among networks with the number of cycles $\ll \sum_{i=3}^{|N|}\left(\genfrac{}{}{0pt}{}{|N|}{i}\right)$, complete grid networks have the lowest skewness. (**d**) The network topologies follow polynomial trendlines with a degree of five. The completeness of network topology means negative skewness.

Figure 5 shows the trajectory of $\mathcal{R}\left(i\right)$ for different network topologies with 8–26 nodes. Two observations are noted. First, the rate of change reaches its maximum at the beginning of the percolation process when the number of disrupted links is approximately equal to 5 for network topologies with no undirected cycle. Similar but not identical, the rate of changes reaches its maximum when the number of disrupted links is approximately 6 for network topologies with one undirected cycle. It means one undirected cycle does not affect the trajectory of $R(i)$, but shifts it forward by one link. Second, as the number of cycles increases, the maximum rate of change shifts, meaning that the maximum uncertainty about the decrease in the network performance would occur later in the percolation process.

### Vulnerability and uncertainty in vulnerability bivariate analysis

**Figure 6:**
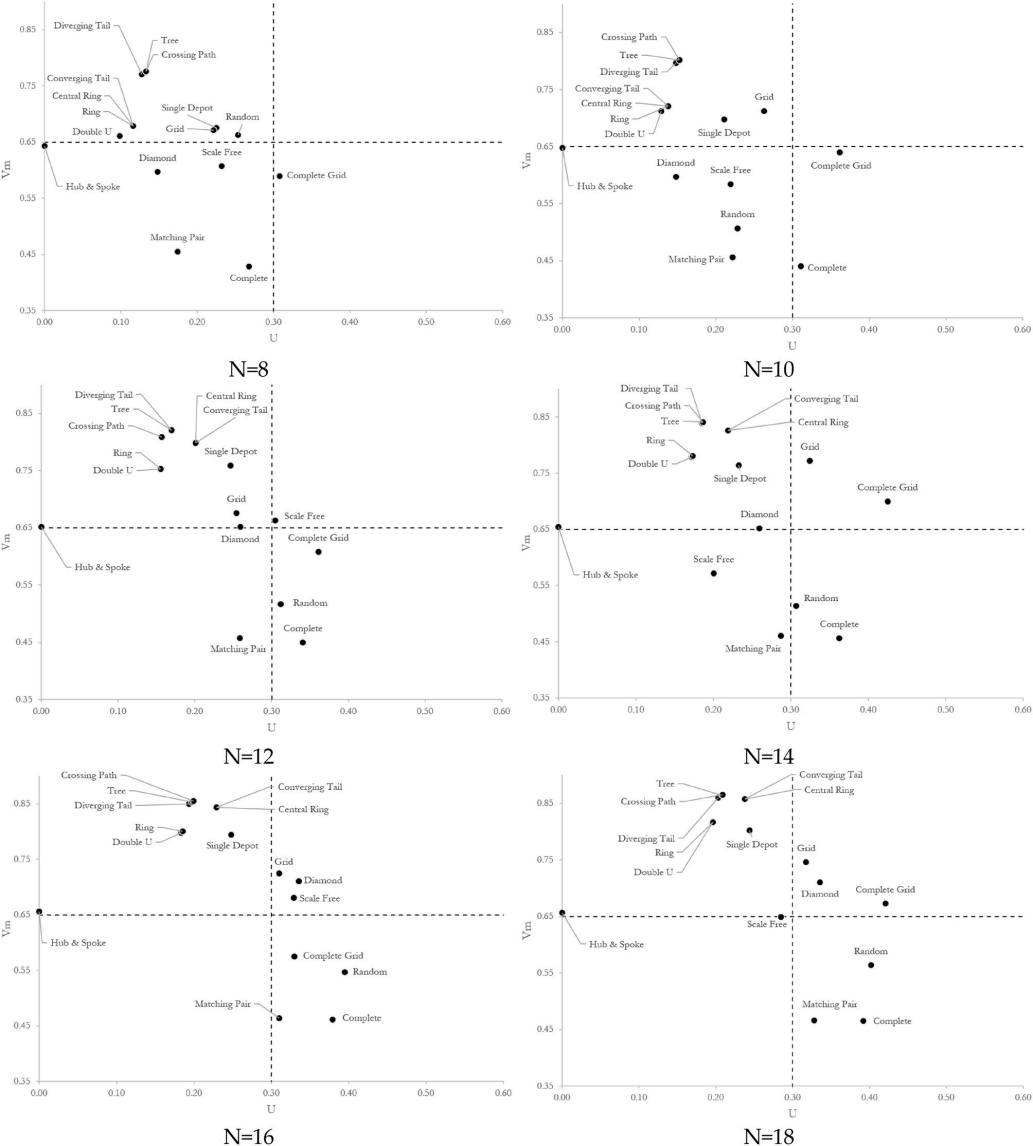

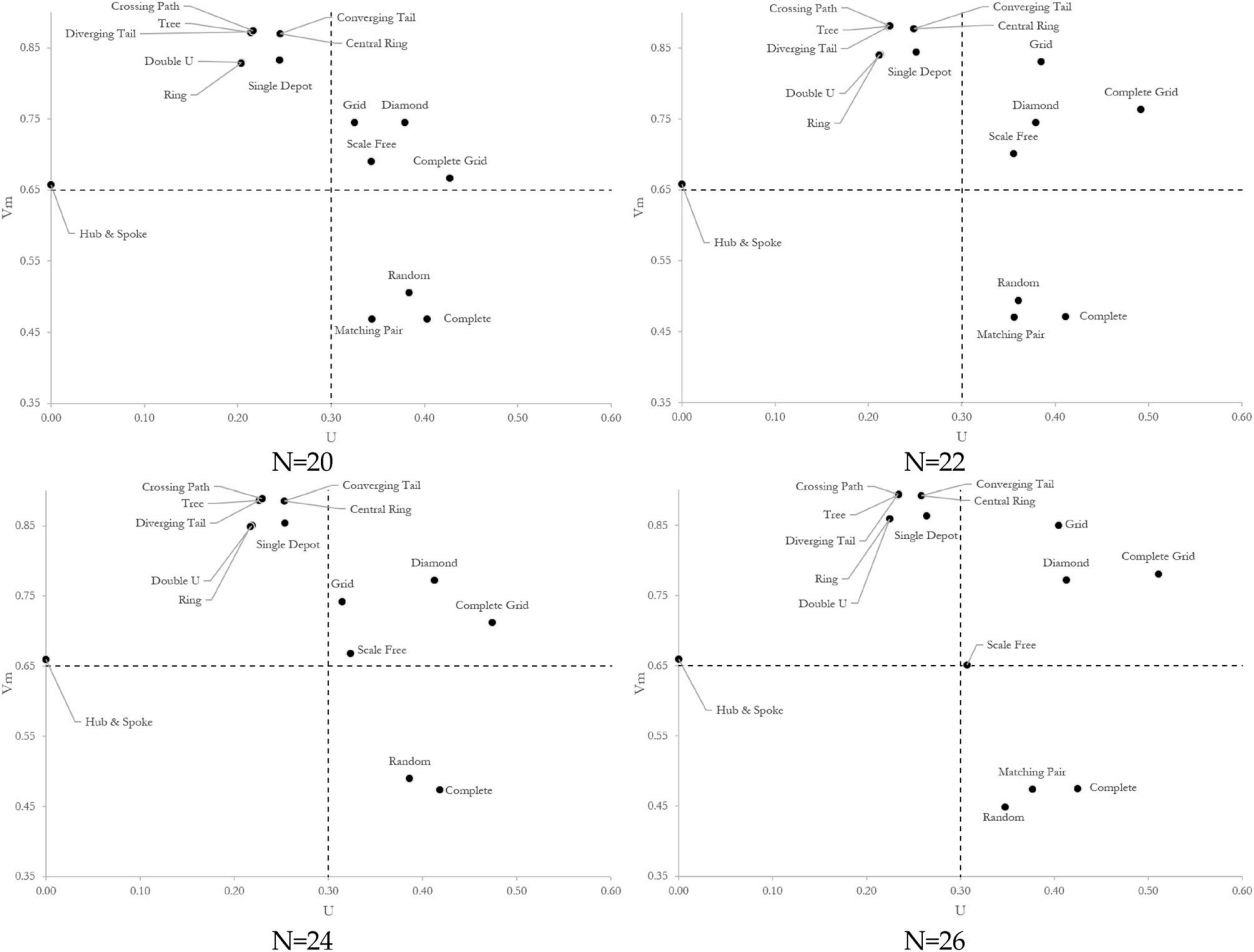
Bivariate analysis between vulnerability and uncertainty in vulnerability measures for different topologies and network sizes. The correlation falls into the top left area with high vulnerability and low uncertainty in vulnerability (HL region) when: (i) the number of cycles equals zero or the network redundancy $\mathcal{R}\mathfrak{e}\left(G\left(N,L\right)\right)=1$, or (ii) the number of cycles equals one or the network redundancy $\mathcal{R}\mathfrak{e}\left(G\left(N,L\right)\right)=\frac{\left|N\right|-1}{\left|N\right|}$. As complete and matching pair networks manifest, the vulnerability measure decreases when the number of cycles increases and the correlation approaches the bottom right area with low vulnerability and high uncertainty in vulnerability (LH region). The decrease in the network size when $\left|N\right|\le 12$, decreases the uncertainty in vulnerability measure and moves the correlation into the bottom left area with low vulnerability and low uncertainty in vulnerability (LL region). However, when $\left|N\right|\ge 14$, the fewer number of links than ${P}_{2}^{N}$, the higher the vulnerability measure of the network, and the correlation falls into the top right area (HH region).

We prove that the vulnerability quantity falls into the interval $\left(\mathrm{0.33,1}\right)$ and the uncertainty in vulnerability quantity falls into the interval $[\mathrm{0,0.66})$, regardless of the network’s topology. The proof is represented in Supplementary Information II. Immediately, we define four domains clustering network topologies with (i) High vulnerability-Low uncertainty in vulnerability (HL), (ii) High vulnerability-High uncertainty in vulnerability (HH), (iii) Low vulnerability-Low uncertainty in vulnerability (LL), and (iv) Low vulnerability-High uncertainty in vulnerability (LH). Figure 6 depicts the results for vulnerability under malicious attack. We draw similar results for vulnerability under benign attacks in Supplementary Information IV*.*

In small-sized networks with 8 nodes, it is observed that all topologies are in either LL or HL, with the complete grid topology as an exception. This means the uncertainty in the vulnerability of networks is low regardless of the topology. In particular, it is observed that (i) diverging tail, crossing path, tree, double u, ring, central ring, converging tail, grid, and random topologies locate in the HL region, (ii) diamond, scale-free, matching pair, single depot, complete, and hub-and-spoke fall in the LL region, and (iii) complete grid resides in the LH region. No network topology is detected in the HH region for networks with 8 nodes. With an increase in the size of the network, a steady increase in both vulnerability and uncertainty in vulnerability is recognized to the extent that no network topology remains in the LL region. The rate of escalation, however, depends on the network topology. The lowest rate goes to hub-and-spoke with a slight increase in vulnerability and no change in uncertainty in vulnerability when there is an increase in the size of the network. The highest shift is noticed for the diamond topology, which moves from the LL region to the HH region.

Diverging tail, crossing path, tree, double u, ring, central ring, converging tail, and single depot remain in the HL region. Still, the vulnerability and uncertainty in vulnerability increase with an increase in the size of the network. Grid, complete grid, and diamond move to the HH region as the network size increases. This shift is due to the ascending increase in the number of links with an increase in the size (i.e., number of nodes) of the network as illustrated by the $\beta$ measure in the following:

- For the grid topology, $\beta =2-\frac{\left|N\right|}{n\left(\left|N\right|-n\right)}$ where $n<\left|N\right|\wedge n\left(\left|N\right|-n\right)=|N|$. As the number of nodes $|N|$ increases from 4 to infinity, $\beta$ increases from 1 to 2, indicating an ascending increase in the number of links.
- The complete grid network topology closely resembles the complete topology. Mathematically, we show the resemblance by measuring the difference between $\beta$ of complete and complete grid topologies. $\Delta \beta ={\beta }_{Complete}-{\beta }_{Grid Complete}=\frac{\left|N\right|\left(\left|N\right|-1\right)}{2\left|N\right|}-\frac{8n\left(\left|N\right|-n\right)-6\left|N\right|}{2\left|N\right|}=\frac{\left|N\right|}{2}-\frac{2-4{n}^{2}}{\left|N\right|}-n+\frac{5}{2} .$ Knowing that $\left(\left|N\right|-n\right)=|N|$ is held for complete grid topologies, $\frac{\partial \Delta \beta }{\partial |N|}={\left|N\right|}^{2}+8\left|N\right|+8\left|N\right|n-4>0, \left|N\right|\ge 2$ demonstrates that the function is strictly ascending and $\frac{{\partial }^{2}\Delta \beta }{\partial {\left|N\right|}^{2}}=\frac{8{n}^{2}-4}{{\left|N\right|}^{3}}>0, n>1$ demonstrates that the function is strictly concave. As the network size goes to infinity, the complete grid topology deviates from the complete topology and approaches the grid topology as $\lim_{\left| N \right| \to \infty } \beta_{Grid Complete} = 4$ and $\lim_{\left| N \right| \to \infty } \beta_{Grid } = 2$.
- For the diamond topology, we notice that the network size increases the number of redundant links by $(k-1)$, where $\left|N\right|=4k-1$, and $\beta =1+\frac{k}{4k-1}$.

A similar pattern is noticed for the scale-free network topology. The scale-free topology switches from LL to the HH region as the network size increases. We speculate that the shift is due to the positive correlation between the number of hubs and the size of the network in the scale-free topology. An increase in the number of hubs increases the disruption scenario alternatives and the uncertainty in vulnerability. Matching pair, complete, and random network topologies move to the LH region as they experience a higher increase in uncertainty in vulnerability than in vulnerability with an increase in the size of the network.

## Discussion

Without in-depth knowledge of the network response under attacks, there is a risk of underestimating the stochasticity of disruptive impacts on the network performance. This might cause a loss of control over the network devolution trajectory, and makes room for unexpected cascading failures like what was induced by a massive winter storm in Texas in 2021. Understanding the complexity of network response under attack calls for a holistic approach to visualize and quantify the vulnerability trajectory of networks when experiencing the loss of performance. We have introduced two quantities of vulnerability $(V)$ and uncertainty in vulnerability $(U)$, and visually examined the transition of complex networks from an original performance state to a complete disruption for sixteen distinct topologies. Our selected topologies exist in biology, economy, sociology, and civil infrastructures. Without loss of generality, we have examined networks with 8 to 26 nodes to ponder the nexus of vulnerability response and the size of the network.

Ranking network topologies by their vulnerability, we have determined crossing path, tree, and diverging tail are the most vulnerable networks. At the same time, complete and matching pairs exhibit almost the lowest vulnerability under the malicious attack. The largest gap in vulnerability exists between tree and complete topologies to the extent that the vulnerability of the tree topology is almost 2 times the complete topology. We have also determined that the vulnerability of networks increases with an increase in the size of the network and notice a slight change in the vulnerability ranking of networks. Ranking network topologies by their uncertainty in vulnerability have indicated a low correlation between our two quantities. *Ipso facto*, uncertainty in vulnerability is not necessarily a function of vulnerability when different topologies are considered. We have recognized that complete grid and complete topologies show the most uncertainty in vulnerability, unlike hub-and-spoke and double u. Similar to the vulnerability, uncertainty in the vulnerability of networks increases with an increase in the size of the network. This finding has practical implications in network inauguration or expansion as it provides insights on (i) how the vulnerability of a network topology changes with an increase in the size and (ii) how the vulnerability of a network can be regulated by shepherding the network growth into the desired topology.

The findings have demonstrated that the vulnerability response of networks to an increase in size falls into two categories depending on the network’s topology. For the first category, vulnerability increases at a faster rate than the corresponding uncertainty in vulnerability. The hub-and-spoke and single depot topologies are the only examples. The uncertainty in vulnerability of the hub-and-spoke topology equals zero regardless of the network size. For the second category, uncertainty in vulnerability increases at a faster rate than the corresponding vulnerability. The remaining network topologies, except the random topology, fall in this category. As suggested by its name, random is the only topology with unstable behavior. Although the uncertainty in vulnerability increases with an increase in the size of random networks, their vulnerability fluctuates and is not a function of the size.

Depending on the network topology, the structure of the residual network may or may not change under percolation. For instance, the tree and ring topologies maintain and lose their configuration, respectively, under percolation in which links are removed at random or in order of their degrees. A tree network with 8 nodes and 7 links shrinks to two tree topologies following a link removal. However, a ring network with 8 nodes and 8 links metamorphoses into a tree network with 7 links following a link removal. This leads the performance loss trajectory of networks to diverge or converge under benign and malicious attacks over the percolation process, forming a heterogeneous uncertainty in vulnerability. It means the area between the upper and lower bounds of performance loss trajectories does not shrink or expand continually when links are removed in order of their degrees. We have argued that the contour of the area is a function of the network topology, and the number of cycles explains the heterogeneous uncertainty in vulnerability formed over the percolation process. In networks with no undirected cycle, the uncertainty in vulnerability is maximum earlier in the percolation process. Diverging tail, tree, and crossing paths are three examples. Interestingly, they also exhibit the highest vulnerability. With an increase in the number of cycles, the uncertainty in vulnerability is accumulated at the end of the percolation process. An extreme example is detected in complete, matching pair, and random topologies. Interestingly, they also exhibit the lowest vulnerability. Hence, we have indicated a negative correlation between the vulnerability and network circuity estimated by $\beta$ measure. It means an increase in the number of cycles reduces the vulnerability of networks. We have tentatively argued that a network is less vulnerable with a considerably high β. On the contrary, the rate of growth in the number of links over the number of nodes $\left(\frac{\partial \beta }{\partial \left|N\right|}\right)$ is positively correlated with the uncertainty in the vulnerability of networks.

This immediately has practical implications. Not only are tree, crossing path, and diverging tail the most vulnerable topologies, but they also exhibit the highest uncertainty in performance loss earlier in the process when they experience a random attack. This emphasizes the time sensitivity of maintenance and recovery of tree, crossing path, and diverging tail topological systems when they are under attack and the role of preparedness to avoid the spread of the attack. We take post disruption casualty evacuation as a pertinent example of implementing these implications in transport networks. Here, the search and rescue teams can identify the affected areas with topologies resembling tree, crossing path, and diverging tail as more probable to contain trapped residents. The reason is that all routes out of the affected areas can be disrupted faster than expected. Relying on such inferences, the teams prioritize the emergency deployment across these areas. Conversely, complete and matching pair networks are the least vulnerable topologies but exhibit a wider range of uncertainty in vulnerability at a later stage under a random attack. This is alarming. We analogize them to dormant volcanoes. They might not cause any concern when they are attacked, but they experience a remarkable performance loss as the attack continues and links become unfunctional one by one. This emphasizes the role of protection during the attack. Protecting transport networks under a limited budget provides another pertinent example of using these inferences in the interest of infrastructure network resilience. Planners and emergency managers can identify the minimum number of paths__ if protected__ that can maintain the transport network performance under random attacks. The optimal protection policy enhances the evacuation procedure and reduces the subsequent humanitarian logistics costs.

Potential avenues remain for future research leading to intriguing additional questions that connect the theoretical explorations in this work to real-world networks in specific domains. First, we have considered networks under uniform demand, while most real-world networks exhibit demand patterns with higher-density axes over both time and space and reflect underlying demand patterns. Although our analysis provides a basis for comparison, it might underestimate or overestimate vulnerability or exaggerate it for links intended for higher flows than links designed to ensure basic access. An extension of this work would consider expanding the notion of uncertainty in vulnerability to incorporate variability in demand patterns (e.g., best case attack under most favorable demand, worst case attack under least favorable demand). Second, we have adopted the linear programming for maximum throughput allocation, as flow allocation rules in most networks tend to follow some sort of optimal principle. In certain networks, however, flow allocation follows significantly different principles, such as user-optimized flow rules in congestible transport networks. Third, we have considered networks with unlimited link capacity, whereas, in practice, capacity constraints often lead to progressive failure. The existence of capacitated links affects the post-disruption flow redistribution and changes the performance degradation trajectory and, consequently, the vulnerability bonds. While the new bounds fall into our proposed vulnerability bounds, their patterns will provide information about the trajectory of uncertainty in the vulnerability of capacitated networks, including the magnitude and number of sudden performance degradations. Fourth, we have produced our analysis under the link percolation assumption. While much of the literature has focused on link percolation, equally important is node percolation, which would debilitate many links simultaneously. This is especially acute for certain topologies such as hub-and-spoke networks, where an attack on hub nodes could make an entire network unfunctional.

## Materials and methods

### Network performance formulation

The proposed formulation maximizes the total delivered demand in the residual network. In a network with $\left|\mathrm{N}\right|$ nodes, there is a total of ${\mathrm{P}}_{2}^{|\mathrm{N}|}=\left|\mathrm{N}\right|\left(\left|\mathrm{N}\right|-1\right)$ origin–destination pairs. Assuming one unit of demand is transferred between each origin–destination pair, the total delivered demand equals $\left|\mathrm{N}\right|\left(\left|\mathrm{N}\right|-1\right)$. More precisely, each node $\mathrm{i}\in \mathrm{N}$ is the origin for each $\left|\mathrm{N}\right|-1$ other nodes sending one unit of demand to each destination. This yields to $\left|\mathrm{N}\right|-1$ units of demand production in total. Each node $\mathrm{i}\in \mathrm{N}$ is also the destination of $\left|\mathrm{N}\right|-1$ other nodes receiving one unit of demand. This yields to $\left|\mathrm{N}\right|-1$ units of demand attraction in total. As a classroom example, consider a ring network with $\left|\mathrm{N}\right|=4$ and $\left|\mathrm{L}\right|=4$. The total origin–destination pairs and the total delivered demand equal 12. The total demand production for each node equals 3, as there are 3 destination nodes for each origin. Similarly, the total demand attraction for each node equals 3, as there are 3 origin nodes for each destination.

As we assume one unit of demand is transferred between each origin–destination pair, the total flow passing each link is the same as its betweenness centrality. We represent $\mathrm{OD}$ as the set of origin–destination pairs, ${\upomega }_{\mathrm{ij}}$ as the total delivered flow from origin $\mathrm{i}\in \mathrm{N}$ to destination $\mathrm{j}\in \mathrm{N}-\{\mathrm{i}\}$, and ${\mathrm{f}}_{\mathrm{ijhk}}$ as the flow of origin–destination pair $\left(\mathrm{i},\mathrm{j}\right)\in \mathrm{OD}$ that passes link $(\mathrm{h},\mathrm{k})\in \mathrm{L}$.

7 $$\begin{gathered} {\text{maximum}} \mathop \sum \limits_{{\left( {\text{i,j}} \right) \in {\text{OD}}}} \left( {{\text{M}}\upomega_{{{\text{ij}}}} - \mathop \sum \limits_{{\left( {\text{h,k}} \right) \in {\text{L}}}} {{\text{f}}}_{{{\text{ijhk}}}} } \right) \hfill \\ {\text{s.t}}{.} \hfill \\ \end{gathered}$$

8 $$\sum_{\mathrm{k}:(\mathrm{h},\mathrm{k})\in \mathrm{L}}{\mathrm{f}}_{\mathrm{ijhk}}-\sum_{\mathrm{k}:\left(\mathrm{k},\mathrm{h}\right)\in \mathrm{L}}{\mathrm{f}}_{\mathrm{ijkh}}\left\{\begin{array}{c}\le 1 \forall \left(\mathrm{i},\mathrm{j}\right)\in OD, h=i \\ =0 \forall \left(\mathrm{i},\mathrm{j}\right)\in OD , h\in N-\{i,j\} \\ ={-\upomega }_{\mathrm{ij}} \forall \left(\mathrm{i},\mathrm{j}\right)\in OD , h=j\end{array}\right.$$

9 $$0\le {\upomega }_{\mathrm{ij}}\le 1 \forall \left(\mathrm{i},\mathrm{j}\right)\in \mathrm{OD}$$

10 $$0\le {\mathrm{f}}_{\mathrm{ijhk}}\left\{\begin{array}{l}\le {\mathrm{P}}_{2}^{\left|\mathrm{N}\right|} \forall \left(\mathrm{i},\mathrm{j}\right)\in OD,\forall \left(\mathrm{h},\mathrm{k}\right)\in L-J\\ \le 0 \forall \left(\mathrm{i},\mathrm{j}\right)\in OD,\forall \left(\mathrm{h},\mathrm{k}\right)\in J \end{array}\right.$$

11 $${\mathrm{\varphi }}_{\left|\mathrm{L}\right|-|\mathrm{J}|}=\sum_{(\mathrm{i},\mathrm{j})\in \mathrm{OD}}{\upomega }_{\mathrm{ij}}$$

Equation (7) represents the objective function and maximizes the total delivered demand through the shortest unblocked paths after eliminating $|\mathrm{J}|$ links. $\mathrm{M}$ is a sufficiently big scalar that assigns high weights to the total delivered flow to prevent feasible but inapplicable solutions. We explain it for a hub-and-spoke network with three nodes and six origin–destination pairs (i.e., $\mathrm{OD}=\{\left(\mathrm{1,2}\right),\left(\mathrm{1,3}\right),\left(\mathrm{2,1}\right),\left(\mathrm{2,3}\right),\left(\mathrm{3,1}\right),(\mathrm{3,2})\}$). The expected maximum delivered demand $\sum_{(\mathrm{i},\mathrm{j})\in \mathrm{OD}}{\upomega }_{\mathrm{ij}}$ equals ${\mathrm{P}}_{2}^{3}=6$, and the total network flow equals $\sum_{(\mathrm{h},\mathrm{k})\in \mathrm{L}}{\mathrm{f}}_{\mathrm{ijhk}}=8$, where the flow on each link in $\mathrm{L}=\{(\mathrm{1,2}),\left(\mathrm{1,3}\right),\left(\mathrm{2,1}\right),\left(\mathrm{2,3}\right),\left(\mathrm{3,1}\right), (\mathrm{3,2})\}$ equals $2$. However, when $\mathrm{M}=1$, the model excludes this solution as it results in a negative objective function $(-2)$. The model returns zero total delivered demand and zero total network flow as the optimal solution, resulting in a better objective function $(0)$. We address it by setting the value of $\mathrm{M}$ as $|\mathrm{L}|$. In our hub-and-spoke network example, the model returns the first solution as optimum with the objective function value of 28.

Equation (8) sends one unit of demand from each origin, passes it through transshipment nodes, and delivers it to each destination. Equation (9) delivers no more than one unit of demand from an origin to a destination, and Eq. (10) enables each operational link $\left(\mathrm{h},\mathrm{k}\right)\in \mathrm{L}-\mathrm{J}$ to pass total network flow at once and disables each eliminated link $\left(\mathrm{h},\mathrm{k}\right)\in \mathrm{J}$ to pass any flow across the network. As the model has no binary or integer variables, its solution time would decrease from exponential to polynomial. Equation (11) calculates the network performance ${\mathrm{\varphi }}_{\left|\mathrm{L}\right|-\left|\mathrm{J}\right|}$, which is the residual network after the elimination of $|\mathrm{J}|$ links. We use the Dijkstra algorithm to find the maximum delivered demands through the shortest unblocked paths for large networks.

### Vulnerability algorithm

Two greedy algorithms are proposed to explore the sequence of link removal procedures that respectively (i) maximizes the total unsatisfactory demand after the removal of each link, also called a malicious attack simulation, and (ii) minimizes the total unsatisfactory demand after the removal of each link, also called a benign attack. The input of each algorithm is the topological characteristics and the flow on each link at the original state. After removing a link, the flow is redistributed through the residual network. The procedure repeats until no link remains undisrupted. The Malicious-Benign Attack Simulation algorithm is as follow:


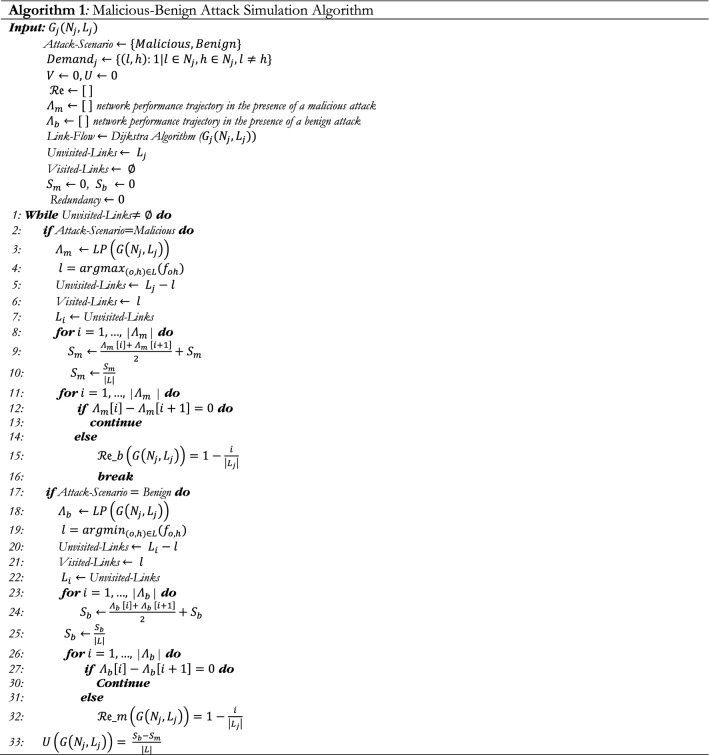


## Supplementary Information

Supplementary Information.

## Data Availability

The datasets used or analyzed during the current study will be available from the corresponding author on reasonable request. Upon the acceptance (1) the authors will post data via a controlled repository and (2) all data will be available for re-use and redistribution, except for commercial use, with proper citations and acknowledgement of the authors.
