# Supplementary material for: Uncertainty in vulnerability of networks under attack
Source: Sci Rep. 2023 Feb 23;13:3179. doi: 10.1038/s41598-023-29899-w (PMC9947912; doi:10.1038/s41598-023-29899-w)
Supplement: Supplementary file 1 — Supplementary Information. [file 41598_2023_29899_MOESM1_ESM.docx]

**Supplementary Information I**

Schematic of 16 network topologies with 12 nodes.

| 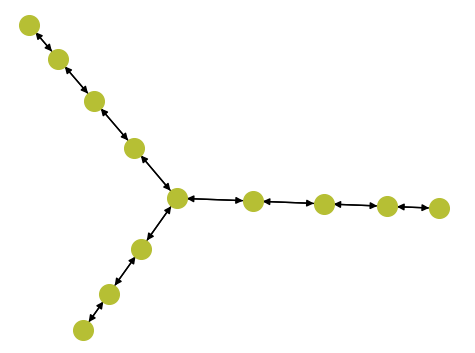 | 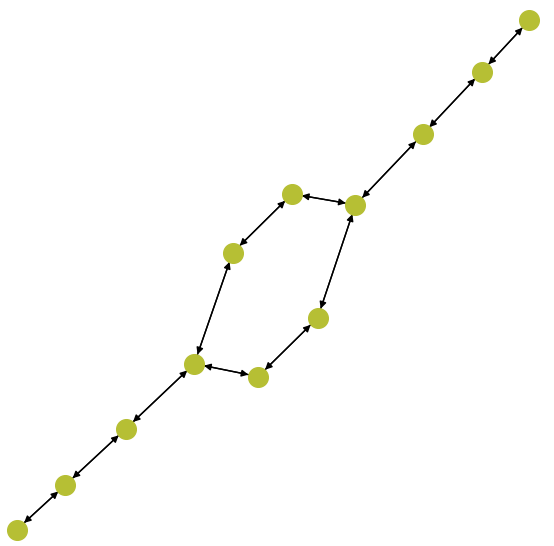 | 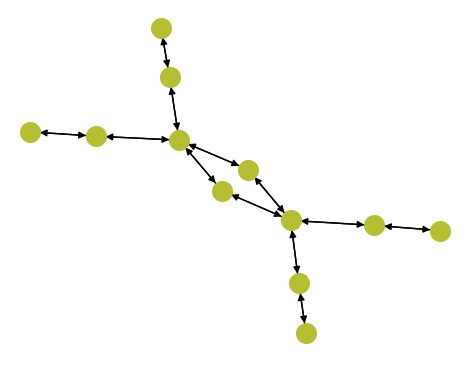 | 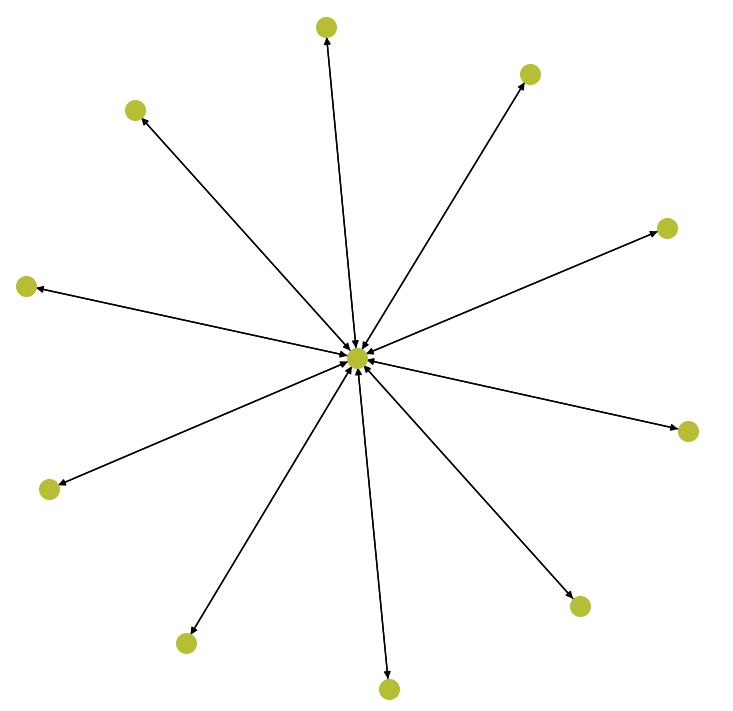 |
| --- | --- | --- | --- |
| Diverging Tail | Converging Tail | Double U | Hub-and-Spoke |
| 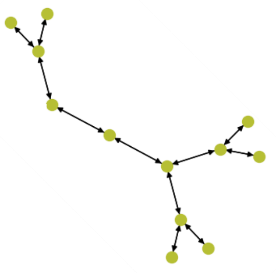 | 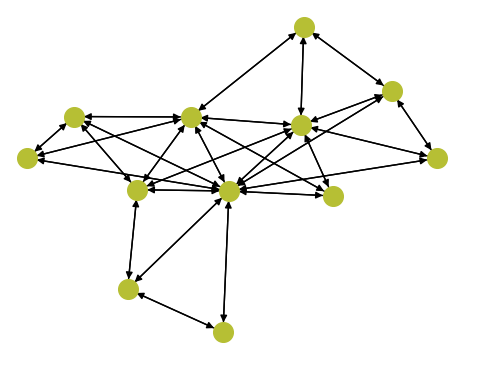 | 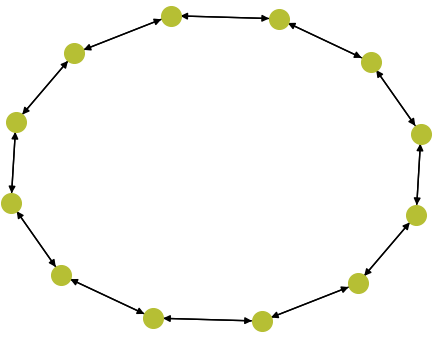 | 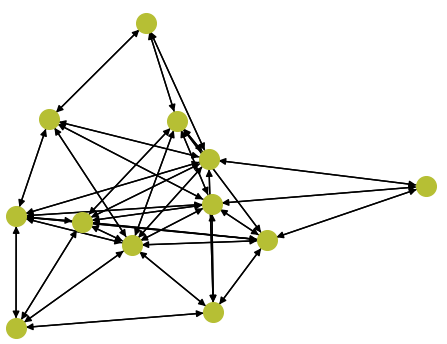 |
| Tree | Scale-Free | Ring | Random |
| 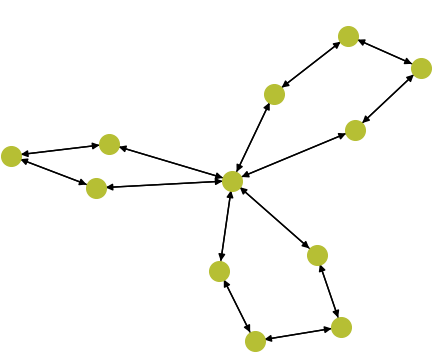 | 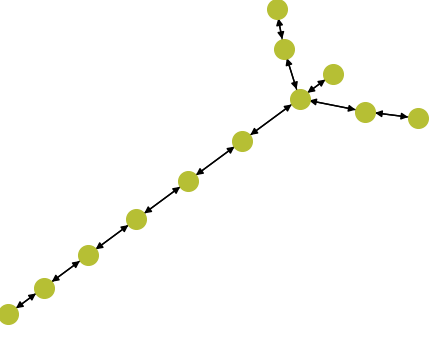 | 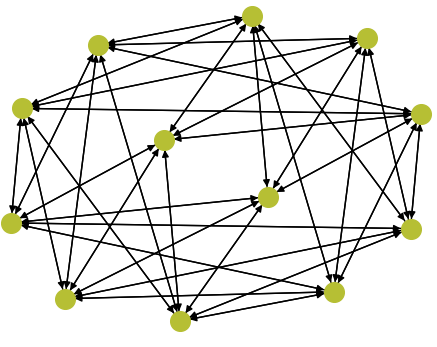 | 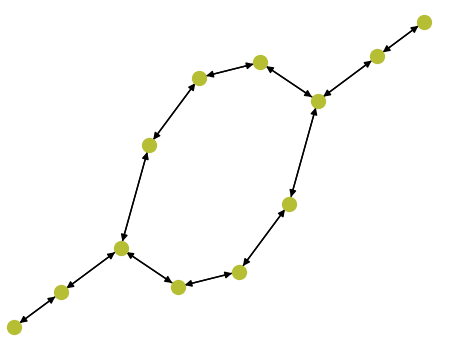 |
| Single Depot | Crossing Path | Matching Pairs | Central Ring |
| 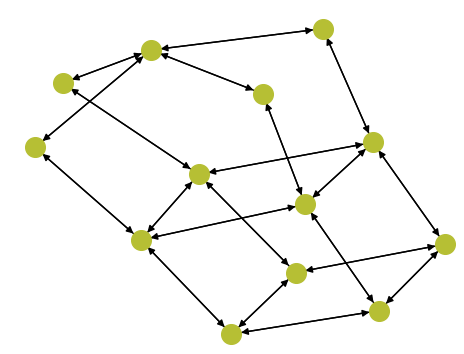 | 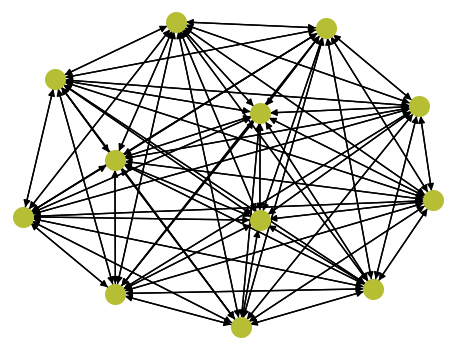 | 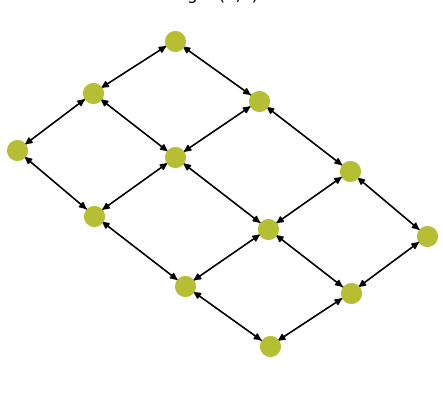 | 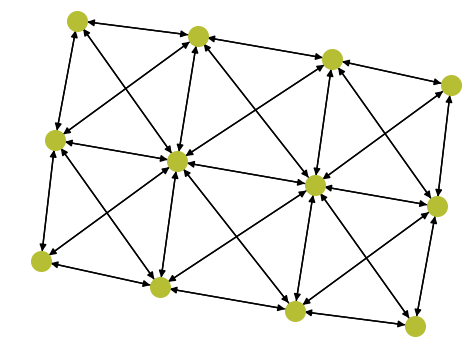 |
| Diamond | Complete | Grid | Complete Grid |

| **FIGURE 1** Schematic of 16 network topologies with 12 nodes. |
| --- |

**Supplementary Information II**

**Global Lower Bound for Network Vulnerability Measure**

The number of parallel paths defines the measure of redundancy $\mathcal{R}\mathfrak{e}\left( G\left( N,L \right) \right)$ and negatively correlates with the network vulnerability measure $V\left( G\left( N,L \right) \right)$. Networks with a higher number of parallel paths (e.g., complete networks) are less vulnerable to catastrophic events. The skewed binary trees are the most vulnerable networks. They include no parallel path (competitive route) as each node connects to at most two neighboring nodes, and the graph must include two nodes with the degree one. We use the vulnerability measure of a skewed tree to introduce a global lower bound for the performance of 16 network topologies.

**Proposition 1.** A skewed undirected tree $G(N,L)$ with $|N|$ nodes and $\left| L \right|=\left| N \right|-1$ links experience the maximum performance degradation after removing Link $\left( i-1,i \right), i\in N, 2<i<|N|$ if the node $i\in N$ is located in the mid-length of the graphs or $i=\left\lfloor\frac{\left| N \right|}{2} \right\rfloor$.

**Proof.** After removing a link from the tree network $G(N,L)$, it breaks into two disconnected residual subgraphs $G^{hk}\left( N_{h}^{k},L_{h}^{k} \right), L_{h}^{k}\subseteq L,N_{h}^{k}\subseteq N, h=1, k=1,2$. Using the proof of contradiction, we first assume that there was another link $\left( j,j+1 \right),j\in N, j\neq i$ that causes maximum network performance degradation if removed. Mathematically, we show the difference in the residual network performance after removing links $\left( i,i+1 \right)$ and $\left( j,j+1 \right)$.

| If we assume $\left( \left\vert N \right\vert-i \right) \left( \left\vert N \right\vert-\left( i+1 \right) \right)+\left( i \right)\left( i-1 \right)>\left( \left\vert N \right\vert-j \right)\left( \left\vert N \right\vert-\left( j+1 \right) \right)+\left( j \right)\left( j-1 \right)$  Then we have $\left\vert N \right\vert^{2}-2\vert N\vert i+2i^{2}-\left\vert N \right\vert>\left\vert N \right\vert^{2}-2\vert N\vert j+2j^{2}-\vert N\vert$  and we conclude that $i\left( i-\left\vert N \right\vert\right)>j\left( j-\left\vert N \right\vert\right)$ | (1) |
| --- | --- |

If $i=\frac{\left| N \right|}{2}, j=1\to\left( \left| N \right|-2 \right)^{2}<0$ contradicts the fact that $\left( \left| N \right|-2 \right)^{2}>0$

If $i=\frac{\left| N \right|}{2}, j=\left| N \right|-1\to\left( \left| N \right|-1 \right)^{2}+1<0$ contradicts with the fact that $\left( \left| N \right|-1 \right)^{2}+1>0$

The minimum of $\left| N \right|^{2}-2\left| N \right|j+2j^{2}-\left| N \right|=\left( |N|-j \right)^{2}+j^{2}$ results in $j=\frac{\partial\left( \left( |N|-j \right)^{2}+j^{2} \right)}{\partial j}=\frac{\left| N \right|}{2}\to0>0$ contradicts the assumption that $i\neq j$

Therefore, removing the link in the middle of a skewed tree causes maximum degradation in the network performance. ■

**Proposition 2.** In a disrupted undirected tree $G^{h}(N,L)$ with $|N|$ nodes and $\left| L \right|_{h}=\left| N \right|-1-h$ links, removing the $h^{th}$ sequential link results in $h+1$ number of subgraphs $G^{hk}\left( N_{h}^{k},L_{h}^{k} \right)$, where $h=1,2,\ldots,\left| N \right|-1$, and $k=1,2,\ldots,h+1$. The network experiences the maximum performance degradation after removing link $\left( i-1,i \right)\in L_{h}, i\in N$ in the middle of the residual subgraph with the maximum of network size, or $i=\left\lfloor\frac{\left| N_{h}^{k} \right|}{2} \right\rfloor,argmax_{G^{hk}\left( N_{h}^{k},L_{h}^{k} \right),k=1,\ldots,h+1}L_{h}^{k}$.

**Proof.** Assuming an undisrupted tree $G(N,L)$, each node sends one unit of demand to each of the $\left| N \right|-1$ nodes. The total satisfied demand equals $|N|(\left| N \right|-1)$. Removing the first link $\left( i-1,i \right), i\in N, 2<i<|N|$ results in two subgraphs $G^{11}\left( N_{1}^{1},L_{1}^{1} \right)$ with $\left| N_{1}^{1} \right|=i-1$ nodes and $\left| L_{1}^{1} \right|=i-2$ links, and $G^{12}\left( N_{1}^{2},L_{1}^{2} \right)$ with $\left| N_{1}^{2} \right|=\left| N \right|-i+1$ nodes and $\left| L_{1}^{2} \right|=\left| N \right|-i$ links.

The residual network performance $\varphi\left( \left| L \right|-1 \right)$ reflects the summation of satisfied demands across the resulted in residual subgraphs $\left( i \right)\left( i-1 \right)+\left( \left| N \right|-i \right)\left( \left| N \right|-i-1 \right)={2i}^{2}-2\left| N \right|i-|N|+\left| N \right|^{2}$.

As the second-order derivation of the network performance function $\frac{\partial^{2}\varphi\left( G\left( N,L_{1} \right) \right)}{\partial i^{2}}=4>0$ the network is strictly convex with one global minimum. The minimum residual network performance happens if the first-order derivative $\frac{\partial\varphi\left( G\left( N,L_{1} \right) \right)}{\partial i}=4i-2\left| N \right|=0\to i=\frac{\left| N \right|}{2}$, or the graph is split in two.

As shown in Figure 1, in each step of $h=2,\ldots,|N|$, the subgraph with the maximum number of links is chosen for the subsequent link removal as it causes maximum network performance degradation. Figure 1 illustrates a disrupted skewed tree with $k<|N|$ number of residual subgraphs.


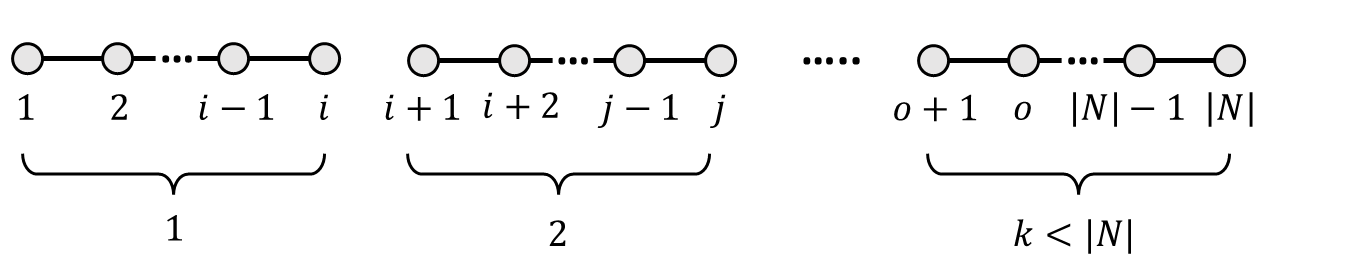


**FIGURE 1** A disrupted skewed tree network $G(N,L_{k-1})$ with $k<|N|$ residual subgraphs.

The total network performance equals $\varphi\left( G\left( N,L_{k-1} \right) \right)=i\left( i-1 \right)+\left( j-i \right)\left( i-j-1 \right)+\ldots+\left( \left| N \right|-o \right)\left( \left| N \right|-o-1 \right)$. The maximum network performance degradation across each subgraph $k=1,2,\ldots,h+1$ happens with the removal of link $\left( \frac{\left( {\eta\left[ 2m+2 \right]}-{\eta\left[ 2m+1 \right]}+1 \right)}{2},\frac{\left( {\eta\left[ 2m+2 \right]}-{\eta\left[ 2m+1 \right]}+1 \right)}{2}+1 \right), \eta=\left[ 1,i,i+1,j,j+1,\ldots,o,o+1,\left| N \right| \right], m=0,1,2,\ldots,h$. $\eta$ is the set of nodes with a degree of 1 or nodes located at the end of each subgraph. $\frac{\left( {\eta\left[ 2m+2 \right]}-{\eta\left[ 2m+1 \right]}+1 \right)}{2}$ is the node located in the middle of the $m^{th}$ subgraph, and ${\eta\left[ 2m+2 \right]}-{\eta\left[ 2m+1 \right]}+1$ is the number of nodes in the $m^{th}$ divided subgraph. Each subgraph experiences degradation of

| $\Delta\varphi(h)=\varphi\left( G\left( N,L_{h} \right) \right)-\varphi\left( G\left( N,L_{h+1} \right) \right)$ | (2) |
| --- | --- |

Here, $\Delta\varphi(h)$ is the network performance degradation after removing one more link from the residual tree network with $h$ disrupted links. $\varphi\left( G\left( N,L_{h} \right) \right)$ is the residual network performance and $L_{h}$ is the set of operational links after removing $h$ links sequentially, and is calculated as:

| $\varphi\left( G\left( N,L_{h} \right) \right)=\sum_{m=1}^{h} \left( {\eta\left[ 2m+2 \right]}-{\eta\left[ 2m+1 \right]}+1 \right)\left( {\eta\left[ 2m+2 \right]}-{\eta\left[ 2m+1 \right]} \right)$ | (3) |
| --- | --- |

$L_{h+1}$ is the set of operational links after removing link $\left( \frac{\left( {\eta\left[ 2m+2 \right]}-{\eta\left[ 2m+1 \right]}+1 \right)}{2}-1,\frac{\left( {\eta\left[ 2m+2 \right]}-{\eta\left[ 2m+1 \right]}+1 \right)}{2} \right)$ from $L_{h}$. Eq. 4 calculates $\varphi(G\left( N,L_{h+1} \right))$ as the residual network performance after removing the corresponding link.

| $\varphi(G\left( N,L_{h+1} \right))=\sum_{m=1}^{l-1<h} \left( {\eta\left[ 2m+2 \right]}-{\eta\left[ 2m+1 \right]}+1 \right)\left( {\eta\left[ 2m+2 \right]}-{\eta\left[ 2m+1 \right]} \right)+\left( {\eta\left[ 2l+2 \right]}-\frac{\left( {\eta\left[ 2l+2 \right]}-{\eta\left[ 2l+1 \right]} \right)}{2}+1 \right)\times\left( {\eta\left[ 2l+2 \right]}-\frac{\left( {\eta\left[ 2l+2 \right]}-{\eta\left[ 2l+1 \right]} \right)}{2} \right)+\left( \frac{\left( {\eta\left[ 2l+2 \right]}-{\eta\left[ 2l+1 \right]} \right)}{2}-\eta\left[ 2l+1 \right] \right)\times\left( \frac{\left( {\eta\left[ 2l+2 \right]}-{\eta\left[ 2l+1 \right]} \right)}{2}-\eta\left[ 2l+1 \right]-1 \right)+\sum_{m=l+1}^{h} \left( {\eta\left[ 2m+2 \right]}-{\eta\left[ 2m+1 \right]}+1 \right)\left( {\eta\left[ 2m+2 \right]}-{\eta\left[ 2m+1 \right]} \right)$ | (4) |
| --- | --- |

We calculate the difference in network performances as:

| $\Delta\varphi\left( h \right)=\left( {\eta\left[ 2l+2 \right]}-{\eta\left[ 2l+1 \right]}+1 \right)\left( {\eta\left[ 2l+2 \right]}-{\eta\left[ 2l+1 \right]} \right)-\left( {\eta\left[ 2l+2 \right]}-\frac{\left( {\eta\left[ 2l+2 \right]}-{\eta\left[ 2l+1 \right]} \right)}{2}+1 \right)\times\left( {\eta\left[ 2l+2 \right]}-\frac{\left( {\eta\left[ 2l+2 \right]}-{\eta\left[ 2l+1 \right]} \right)}{2} \right)-\left( \frac{\left( {\eta\left[ 2l+2 \right]}-{\eta\left[ 2l+1 \right]} \right)}{2}-\eta\left[ 2l+1 \right] \right)\times\left( \frac{\left( {\eta\left[ 2l+2 \right]}-{\eta\left[ 2l+1 \right]} \right)}{2}-\eta\left[ 2l+1 \right]-1 \right)$ | (5) |
| --- | --- |

We simplify Eq. (5) as:

| $\Delta\varphi\left( h \right)={\eta\left[ 2l+2 \right]}-{\eta\left[ 2l+1 \right]}-4{{\eta\left[ 2l+1 \right]}}^{2}$ | (6) |
| --- | --- |

Eq. (6) proves that the performance of each residual subgraph has a positive correlation with the distance between two tails of the network. Dividing a subgraph with a larger size in half decreases the network performance more than dividing its counterpart network in a smaller size. ■

Figure 2 illustrates how to calculate the lower bound vulnerability for network topologies with size $N$. We consider a skewed tree with size $N$, and divide the subgraph in two by removing the link in the middle. In each step, $h=1,2,\ldots,\left| N \right|-1$, the subgraph with the maximum size is chosen for the link removal procedure. For each $h=1,\ldots,\left| N \right|-1$, the index $l=1,2,\ldots,h$ in the denominator of $\frac{\left| N \right|}{2^{l}}$ changes aligned with the configuration of $h$:

If $h=2k\Rightarrow l=h$, and if $h=2k+1\Rightarrow l=h-1$.


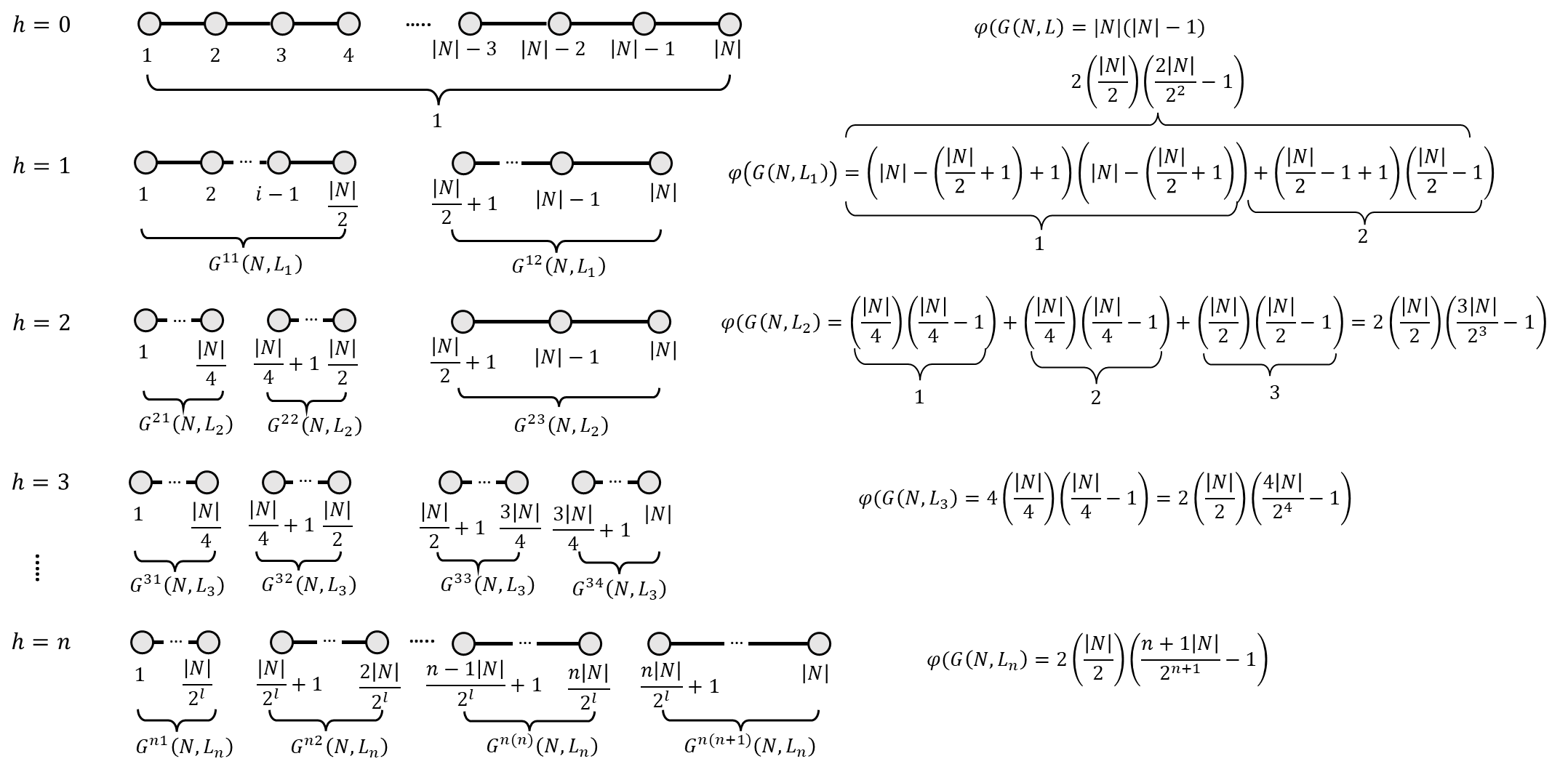


**FIGURE 2** Skewed tree network performance trajectory under malicious attacks.

The normalized network performance after the disruption of $h^{th}$ link is

| $\Lambda\left( h \right)=\frac{\varphi\left( G\left( N,L_{h} \right) \right)}{\varphi\left( G\left( N,L \right) \right)}=\frac{2\left( \frac{\left\vert N \right\vert}{2} \right)\left( \frac{\left( h+1 \right)\left\vert N \right\vert}{2^{h+1}}-1 \right)}{\left\vert N \right\vert\left( \left\vert N \right\vert-1 \right)}=\frac{\frac{\left( h+1 \right)\left\vert N \right\vert}{2^{h+1}}-1}{\left\vert N \right\vert-1}$ | (7) |
| --- | --- |

The area under the network performance degradation trajectory is:

| $S_{m}=\frac{1}{2}\left( \sum_{h=0}^{\left\vert N \right\vert-2} \Lambda\left( h \right)+\Lambda\left( h+1 \right) \right)=\frac{1}{2}\left( \frac{3\left\vert N \right\vert-4}{2\left\vert N \right\vert-2}+\sum_{h=1}^{\left\vert N \right\vert-2} \frac{\frac{\left( h+1 \right)\left\vert N \right\vert}{2^{h+1}}-1}{\left\vert N \right\vert-1}+\frac{\frac{\left( h+2 \right)\left\vert N \right\vert}{2^{h+2}}-1}{\left\vert N \right\vert-1} \right)$ | (8) |
| --- | --- |

the skewed tree network vulnerability is:

| $V\left( G\left( N,L \right) \right)=1-\frac{S_{m}}{\left\vert L \right\vert}= 1-\frac{1}{2}\times\frac{\frac{3\left\vert N \right\vert-4}{2}+\sum_{h=1}^{\left\vert N \right\vert-2} \frac{\left( 3h+4 \right)\left\vert N \right\vert}{2^{h+2}}-2}{{{\left( \left\vert N \right\vert-1 \right)^{2}}}}$ | (9) |
| --- | --- |

If the network size increases to infinity, the limit of the network vulnerability approach:

| $\lim_{\left\vert N \right\vert\to\infty} V\left( G\left( N,L \right) \right) =1-\frac{1}{2}\lim_{\left\vert N \right\vert\to\infty} \left( \frac{\frac{3\left\vert N \right\vert-4}{2}}{\left( \left\vert N \right\vert-1 \right)^{2}}+\frac{\sum_{h=1}^{\left\vert N \right\vert-2} \frac{\left( 3h+4 \right)\left\vert N \right\vert}{2^{h+2}}}{{{\left( \left\vert N \right\vert-1 \right)^{2}}}}-\frac{2\left( \left\vert N \right\vert-2 \right)}{\left( \left\vert N \right\vert-1 \right)^{2}} \right)$ | (10) |
| --- | --- |

Using the geometric series conversions $\sum_{h=1}^{\infty} \frac{h}{2^{h}}=2$, and $\sum_{h=1}^{\infty} \frac{1}{2^{h}}=1$, we have:

| $\lim_{\left\vert N \right\vert\to\infty} V\left( G\left( N,L \right) \right) =1-\frac{1}{2}\lim_{\left\vert N \right\vert\to\infty} \frac{\frac{5}{2}\left\vert N \right\vert}{\left( \left\vert N \right\vert-1 \right)^{2}} =1$ | (11) |
| --- | --- |

**Proposition 3.** A skewed tree network experiences the minimum performance degradation if the subgraph with the minimum size is chosen for the subsequent link removal. In addition, the corresponding subgraph experiences minimum performance degradation if it loses a link connecting to a node with degree one.

**Proof.** After removing a link from subgraph $G^{kh}$ $k=1,\ldots,h+1$ in step $h=1,\ldots,\left| N \right|-1$, Eq. (12), the network degradation indicates the polynomial inverse relationship between the subgraph size and network performance degradation. $L_{h+(1)}^{k}$ indicates removing one link, (1), from subgraph $G^{kh}$ of a disrupted graph $G(N,L_{h})$ with $\left| L_{h} \right|=\left| L \right|-h$ residual links.

| $\varphi\left( {G^{kh+1}}\left( N_{h+1}^{k},L_{h+\left( 1 \right)}^{k} \right) \right)=\left\vert N_{h}^{k} \right\vert\left( \left\vert N_{h}^{k} \right\vert-1 \right)-2\left( \frac{\left\vert N_{h}^{k} \right\vert}{2} \right)\left( \frac{2\left\vert N_{h}^{k} \right\vert}{{2^{2}}}-1 \right)=\left( \frac{\left\vert N_{h}^{k} \right\vert^{2}}{2} \right)$  $k=1,\ldots, h+1, h=1,\ldots,\left\vert N \right\vert-1$ | (12) |
| --- | --- |

Aligned with this proof, removing a link with a node of degree one (corner links) degrades the performance of the subgraph to:

| $\varphi\left( {G^{kh+1}}\left( N_{h+1}^{k},L_{h+\left( 1 \right)}^{k} \right) \right)=\left\vert N_{h}^{k} \right\vert\left( \left\vert N_{h}^{k} \right\vert-1 \right)-\left( \left\vert N_{h}^{k} \right\vert-1 \right)\left( \left\vert N_{h}^{k} \right\vert-2 \right)=\left( \left\vert N_{h}^{k} \right\vert+1 \right)$  $k=1,\ldots, h+1, h=1,\ldots,\left\vert N \right\vert-1$ | (13) |
| --- | --- |

Choosing any other link $\left( i,i+1 \right), 2<i<|N|-2$ than the corner links results in the total network degradation of:

| $\varphi\left( {G^{kh}}\left( {N_{h}}^{k},L_{h+\left( 1 \right)}^{k} \right) \right)=\left\vert{N_{h}}^{k} \right\vert\left( \left\vert{N_{h}}^{k} \right\vert-1 \right)-\left( \left( \left\vert{N_{h}}^{k} \right\vert-i \right)\left( \left\vert{N_{h}}^{k} \right\vert-i-1 \right)+\left( i \right)\left( i-1 \right) \right)=2i\left( \left\vert{N_{h}}^{k} \right\vert-i \right)$ | (14) |
| --- | --- |

Employing proof of contradiction, we assume there is a $2<i<\left| {N_{h}}^{k} \right|-2$ where:

| $2i\left( \left\vert{N_{h}}^{k} \right\vert-i \right)<\left( \left\vert{N_{h}}^{k} \right\vert+1 \right)\to\left\vert{N_{h}}^{k} \right\vert\left( 2i-1 \right)<i+1\to\left\vert{N_{h}}^{k} \right\vert<\frac{i+1}{2i-1}$ | (15) |
| --- | --- |

As $\frac{i+1}{2i-1}$ is a strictly decreasing function for $2<i<\left| {N_{h}}^{k} \right|-2$, we have $1<\frac{i+1}{2i-1}<\lim_{\left| {N_{h}}^{k} \right|-2\to\infty} \frac{\left| {N_{h}}^{k} \right|-1}{2\left| {N_{h}}^{k} \right|-5}\approx\frac{1}{2}\Rightarrow\left| N^{k} \right|<1$ that contradicts $\left| {N_{h}}^{k} \right|>1$.■

The normalized network performance after the disruption of $h^{th}$ link is:

| $\Lambda\left( h \right)=\frac{\varphi\left( G\left( N,L_{h} \right) \right)}{\varphi\left( G\left( N,L \right) \right)}=\frac{(\left\vert N \right\vert-h)(\left\vert N \right\vert-h-1)}{\left\vert N \right\vert\left( \left\vert N \right\vert-1 \right)} h=0,1,\ldots,\left\vert N \right\vert-1$ | (16) |
| --- | --- |

And the area under the network performance degradation trajectory is:

| $S_{m}=\frac{1}{2}\left( \sum_{h=0}^{\left\vert N \right\vert-2} \Lambda\left( h \right)+\Lambda\left( h+1 \right) \right)=\sum_{h=0}^{\left\vert N \right\vert-2} \frac{\left( \left\vert N \right\vert-h-1 \right)^{2}}{\vert N\vert(\left\vert N \right\vert-1)}$ | (17) |
| --- | --- |

the skewed tree network vulnerability is:

| $V\left( G\left( N,L \right) \right)=1-\frac{S_{m}}{\left\vert L \right\vert}= 1-\frac{\sum_{h=0}^{\left\vert N \right\vert-2} \left( \left\vert N \right\vert-h-1 \right)^{2}}{\left\vert N \right\vert{\left( \left\vert N \right\vert-1 \right)^{2}}}$ | (18) |
| --- | --- |

If the network size increases to infinity, the limit of the network vulnerability approach:

| $\lim_{\left\vert N \right\vert\to\infty} V\left( G\left( N,L \right) \right) =\lim_{\left\vert N \right\vert\to\infty}1-\frac{1}{6}\times\frac{4\left\vert N \right\vert-3}{\left\vert N \right\vert-1}=\frac{1}{3}$ | (19) |
| --- | --- |

**Global Upper Bound for Network Vulnerability Measure**

Complete networks are severely connected as they have the maximum number of redundant paths among the 16 network topologies. Hence, their performance trajectory under benign attacks is a good candidate to calculate the upper bound for the vulnerability of 16 network topologies.

**Proposition 4.** Complete network $G\left( N,L \right)$ experiences no performance degradation in each step of the sequential link removal process If $\exists i, k_{i}=\left| N \right|-1$ and any link $\left( n,m \right)\in L, n\neq m\neq i$ is chosen for the subsequent link removal procedure. $k_{i}$ is the degree of node $i$.

**Proof.** If there is a node connected to $\left| N \right|-1$ other nodes, the network stays connected as at least one path connects each pair of nodes. ■

Keeping one node $i\in N$ with $k_{i}=\left| N \right|-1$, the network performance remains unchanged during the sequential removal of $\frac{1}{2}\left( \left| N \right|-1 \right)\left( \left| N \right|-2 \right)$. The sequential removal of the $h^{th}$ link degrades the normalized network performance from one to $\varphi(G\left( N,L_{h} \right)=\frac{\left( \left| N \right|-h \right)\left( \left| N \right|-h-1 \right)}{2}, h=\frac{1}{2}\left( \left| N \right|-1 \right)\left( |N|-2 \right)+1,\ldots,\frac{1}{2}\left| N \right|\left( \left| N \right|-1 \right)$.

The normalized network performance after the disruption of $h^{th}$ link is:

| $\Lambda\left( h \right)=\left\{ \begin{matrix} 1 0\leq h\leq\frac{1}{2}\left( \left\vert N \right\vert-1 \right)\left( \vert N\vert-2 \right) \\ \frac{(\left\vert N \right\vert-h)(\left\vert N \right\vert-h-1)}{\left\vert N \right\vert\left( \left\vert N \right\vert-1 \right)} \frac{1}{2}\left( \left\vert N \right\vert-1 \right)\left( \left\vert N \right\vert-2 \right)+1\leq h\leq\frac{\left\vert N \right\vert\left( \left\vert N \right\vert-1 \right)}{2} \end{matrix} \right.$ | (20) |
| --- | --- |

And the area under the network performance degradation trajectory is:

| $S_{b}=\frac{1}{2}\left( \sum_{h=0}^{\frac{\left\vert N \right\vert\left( \left\vert N \right\vert-1 \right)}{2}-1} \Lambda\left( h \right)+\Lambda\left( h+1 \right) \right)=\left( \left\vert N \right\vert-1 \right)\left( \vert N\vert-2 \right)+2\sum_{h=\frac{1}{2}\left( \left\vert N \right\vert-1 \right)\left( \left\vert N \right\vert-2 \right)+1}^{\frac{\left\vert N \right\vert\left( \left\vert N \right\vert-1 \right)}{2}} \frac{\left( \left\vert N \right\vert-h-1 \right)^{2}}{\vert N\vert(\left\vert N \right\vert-1)}$ | (21) |
| --- | --- |

Note that $\sum_{\frac{1}{2}\left( \left| N \right|-1 \right)\left( \left| N \right|-2 \right)+1}^{\frac{\left| N \right|\left( \left| N \right|-1 \right)}{2}} \frac{\left( \left| N \right|-h-1 \right)^{2}}{|N|(\left| N \right|-1)}=\sum_{h=0}^{\left| N \right|-1} \frac{\left( \left| N \right|-h-1 \right)^{2}}{|N|(\left| N \right|-1)}$.

The complete network vulnerability is

| $V\left( G\left( N,L \right) \right)=1-\frac{S_{b}}{\left\vert L \right\vert}= 1-\frac{\left\vert N \right\vert\left( \left\vert N \right\vert-1 \right)^{2}\left( \vert N\vert-2 \right)+\sum_{h=0}^{\left\vert N \right\vert-2} \left( \left\vert N \right\vert-h-1 \right)^{2}}{\frac{1}{2}\left\vert N \right\vert^{2}{\left( \left\vert N \right\vert-1 \right)^{2}}}$ | (22) |
| --- | --- |

If the network size increases to infinity, the limit of the network vulnerability approaches to

| $\lim_{\left\vert N \right\vert\to\infty} V\left( G\left( N,L \right) \right) =\lim_{\left\vert N \right\vert\to\infty}1-\frac{\vert{N\vert}^{4}}{\left\vert N \right\vert^{4}}=0$ | (23) |
| --- | --- |

**Proposition 5.** A complete network $G\left( N,L \right)$ experiences the maximum performance degradation in the sequential link removal procedure if all links $(i,j)\in L$ connected to the node $i\in N$ with the minimum degree $k_{i}=\min_{j\in N} k_{j},k_{j}>1$ are chosen to be removed sequentially. The links are sorted according to the degree of the node $k_{j}, j\in N, (i,j)\in L$, ascendingly.

**Proof.** As there are $\sum_{k=0}^{\left| N \right|-2} \frac{\left( \left| N \right|-2 \right)!}{\left( \left| N \right|-2-k \right)!}=\left( \left| N \right|-2 \right)!\sum_{k=0}^{\left| N \right|-2} \frac{1}{k!}\approx\left( \left| N \right|-2 \right)!e$ number of paths between each pair of nodes in a complete graph, it requires several link removal iterations to disrupt all paths between a pair of nodes completely. However, isolating a node with the minimum node degree is the fastest way to see the network degradation. The beginning of the sequential link removal process takes $|N|-1$ links to isolate a node with the degree of $|N|-1$. The residual network is a disconnected node and a complete subgraph that includes nodes with the degree of $\left| N \right|-2$. The same procedure is repeated until the residual network is $|N|$ disconnected nodes. ■

Following Proposition 4, The network performance changes:

| $\Lambda\left( h_{i} \right)=\frac{\varphi\left( G\left( N,L_{h} \right) \right)}{\varphi\left( G\left( N,L \right) \right)}=\frac{(\left\vert N \right\vert-i)(\left\vert N \right\vert-\left( i+1 \right))}{\left\vert N \right\vert(\left\vert N \right\vert-1)} \left( i-1 \right)\left\vert N \right\vert-\frac{i\left( i-1 \right)}{2}\leq h<\left( i \right)\left\vert N \right\vert-\frac{i\left( i+1 \right)}{2}, i=1,\ldots,\left\vert N \right\vert-1$ | (24) |
| --- | --- |

The area under the network performance degradation trajectory is:

| $S_{m}=\sum_{i=0}^{\left\vert N \right\vert-1} \Lambda\left( h_{i} \right)\left( \left\vert N \right\vert-(i+2) \right)+\frac{1}{2}\left( \sum_{i=0}^{\left\vert N \right\vert-1} \Lambda\left( h_{i} \right)+\Lambda\left( h_{i+1} \right) \right)=\sum_{i=0}^{\left\vert N \right\vert-1} \left( \frac{\left( \left\vert N \right\vert-i \right)\left( \left\vert N \right\vert-\left( i+1 \right) \right)\left( \left\vert N \right\vert-\left( i+2 \right) \right)}{\left\vert N \right\vert\left( \left\vert N \right\vert-1 \right)}+\frac{\left( \left\vert N \right\vert-\left( i+1 \right) \right)^{2}}{\left\vert N \right\vert\left( \left\vert N \right\vert-1 \right)} \right)$ | (25) |
| --- | --- |

Using the geometric series, the complete network vulnerability is:

| $V\left( G\left( N,L \right) \right)=1-\frac{S_{m}}{\left\vert L \right\vert}= 1-\frac{1}{2}\sum_{i=0}^{\left\vert N \right\vert-1} \left( \frac{\left( \left\vert N \right\vert-i-1 \right)\left( \left( \left\vert N \right\vert-\left( i+1 \right) \right)^{2}-(i-1) \right)}{\left\vert N \right\vert^{2}\left( \left\vert N \right\vert-1 \right)^{2}} \right)$ | (26) |
| --- | --- |

If the network size increases to infinity, the limit of the network vulnerability approach:

| $\lim_{\left\vert N \right\vert\to\infty} V\left( G\left( N,L \right) \right) \approx\lim_{\left\vert N \right\vert\to\infty}1-\frac{\frac{1}{4}\left\vert N \right\vert^{4}}{\frac{1}{2}\left\vert N \right\vert^{4}}=\frac{1}{2}$ | (27) |
| --- | --- |

As the network size goes to infinity ($\left| N \right|\to\infty$), the uncertainty measure of the complete network goes to $0.5,$ and the uncertainty measure of the skewed network goes to $0.66$. Mathematically, the difference between the vulnerability measures under benign and malicious attacks defines its uncertainty measure.

| $V_{m}\left( G\left( N,L \right) \right)- V_{b}\left( G\left( N,L \right) \right)=\left( 1- \frac{S_{m}}{\left\vert L \right\vert} \right)-\left( 1- \frac{S_{b}}{\left\vert L \right\vert} \right)=\frac{S_{b}-S_{m}}{\left\vert L \right\vert}=U\left( G\left( N,L \right) \right)$ | (28) |
| --- | --- |

**Proposition 6.** The global upper bound for the network uncertainty measure goes to $0.66$ as the network size goes to infinity.

**Proof.** Skewed tree represents the networks with at most one undirected cycle (e.g., converging tail, diverging tail, crossing path, hub-and-spoke, central ring, ring, double u). Complete network topology represents networks with more undirected cycles (e.g., scale-free, random, matching pair, grid, grid-complete, and diamond graphs).

During the sequential disruption process, acyclic networks (e.g., tree, converging tail, diverging tail, crossing path) behave analogously to the skewed tree network with the same size. They are the most vulnerable if they lose the middle link in the largest residual subgraph and the least vulnerable if they lose the corner links. In networks with one undirected cycle (e.g., ring, central ring, double u) each node is accessible through two parallel paths and adds one unit to the area of network performance trajectory under malicious and benign attacks. For graph $G(N,L)$ representing any of the network topologies we have:

| $1+S_{M}^{G_{skewed tree}}\approx S_{M}^{G}$  $1+S_{B}^{G_{skewed tree}}\approx S_{B}^{G}$ | (29) |
| --- | --- |

Eq. (29) elucidates the role of one redundant path in increasing network robustness. For the networks with one undirect cycle, the vulnerability measures under benign and malicious attacks equal those of the skewed tree network when $|N|$ goes to infinity. Similar to the skewed tree network, the upper bound of the uncertainty measure for those network topologies equals 0.66.

Similar but not identical, the scale-free, random, matching pair, grid, grid-compete, and diamond networks behavior before disruptions is more analogous to that of a complete network than a skewed tree network. The vulnerability of complete networks under benign attacks is the maximum among the networks as it includes the maximum number of a cycle possible in any network topologies, or $\sum_{i=2}^{|N|} \left( \begin{matrix} |N| \\ i \end{matrix} \right)=2^{\left| N \right|}-\left( \left| N \right|+1 \right)$. Under a malicious attack, the vulnerability of each network is higher than the complete network.

Without loss of generality, we consider the root-tree network’s vulnerability measure as the worst-case lower bound for the scale-free network of the same size. The proposed rooted tree network includes one root with a degree of three and nodes with a degree of at most two. The vulnerability measure of ring networks is a worst-case lower bound for the random networks. Eq. (30) and Eq. (31) calculate the vulnerability of the rooted-tree and lattice networks under malicious attacks.

| $V\left( G_{Ring}\left( N,L \right) \right)=1-\frac{1+\left( \sum_{h=0}^{\left\vert N \right\vert-2} \frac{\left( \left\vert N \right\vert-h-1 \right)^{2}}{\left\vert N \right\vert\left( \left\vert N \right\vert-1 \right)} \right)}{\left\vert N \right\vert}=V\left( G_{Skewed Tree}\left( N,L \right) \right)-\frac{1}{\left\vert N \right\vert}$ | (30) |
| --- | --- |

| $V\left( G_{Rooted-Tree}\left( N,L \right) \right)=1-\frac{\left( \sum_{h=0}^{\left\vert N \right\vert-2} \frac{\left( \left\vert N \right\vert-h-1 \right)^{2}}{\left( \left\vert N \right\vert\right)\left( \left\vert N \right\vert-1 \right)} \right)+}{\left\vert N \right\vert}=V\left( G_{Skewed Tree}\left( N,L \right) \right)$ | (31) |
| --- | --- |

If $|N|$ goes to infinity, the vulnerability measure for both ring and rooted-tree network, and consequently, the lower bound vulnerability for each scale-free and random network goes to one. The uncertainty measuring the upper bound for these three networks does not exceed 0.66 as $|N|$ goes to infinity. ■

**Supplementary Information III**

Disruption trajectory of 16 network topologies with 10, 12, 14, 16, 18, 20, 22, 24, and 26 nodes.

| **Diverging Tail** | **Tree** | **Crossing Path** | **Hub-and-Spoke** |
| --- | --- | --- | --- |
| 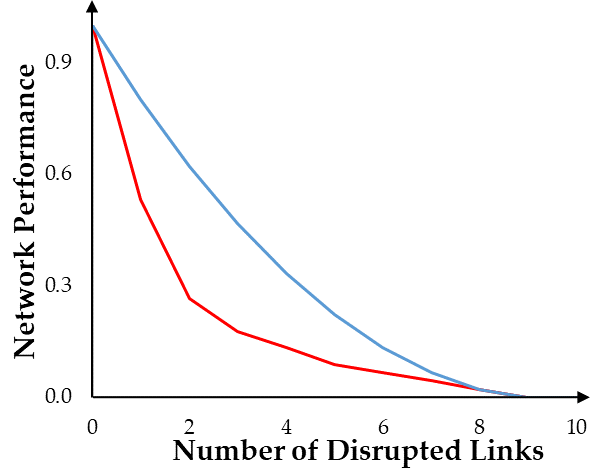 | 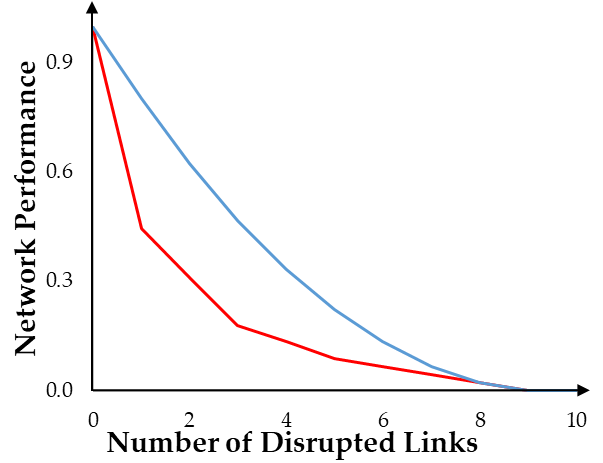 | 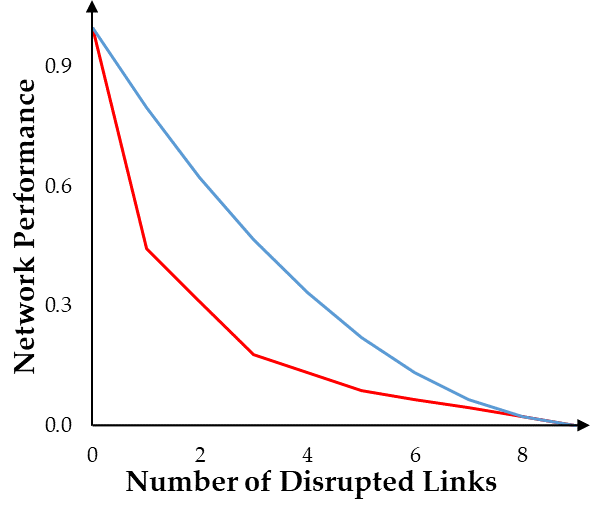 | 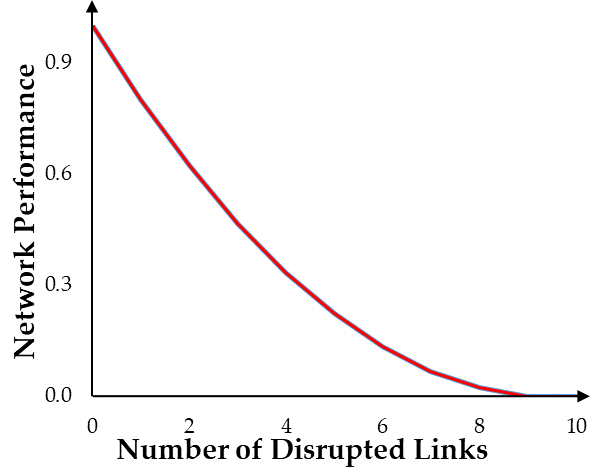 |
| **Central Ring** | **Converging Tail** | **Double U** | **Ring** |
| 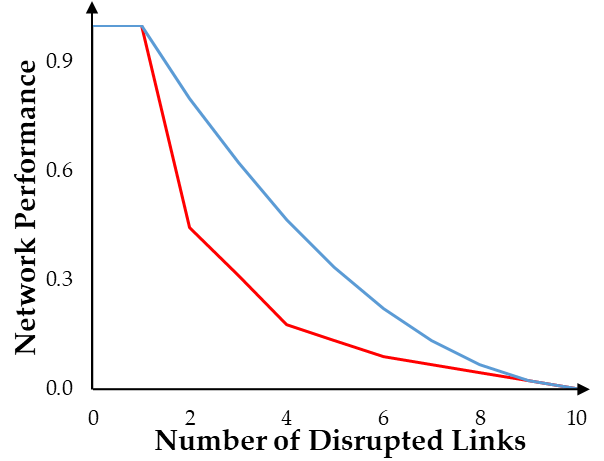 | 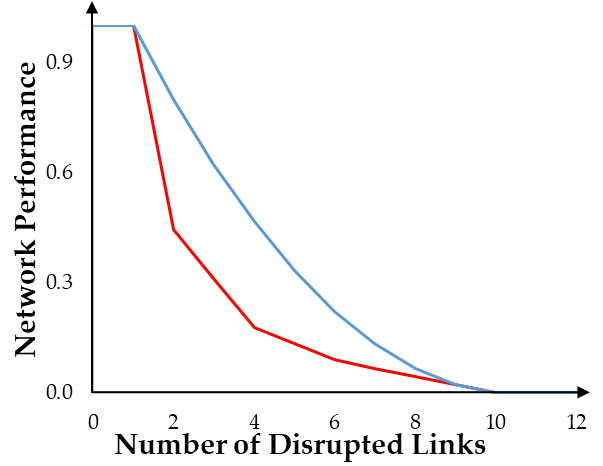 | 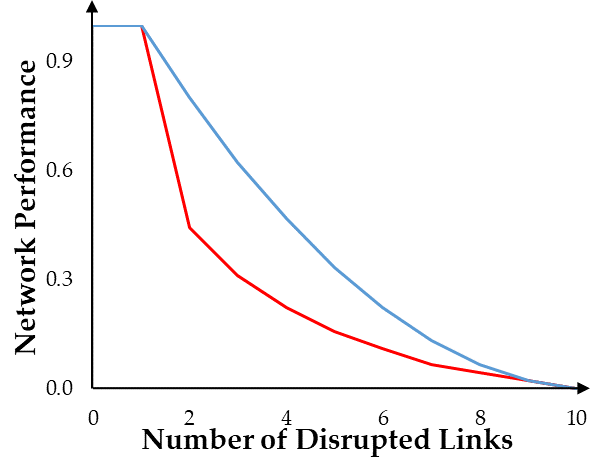 | 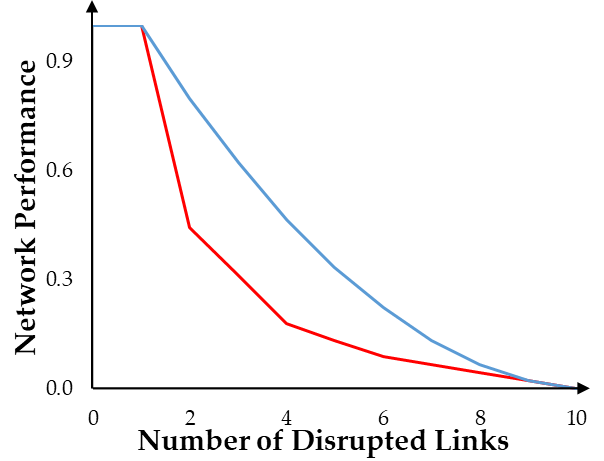 |
| **Single Depot** | **Complete** | **Matching Pairs** | **Diamond** |
| 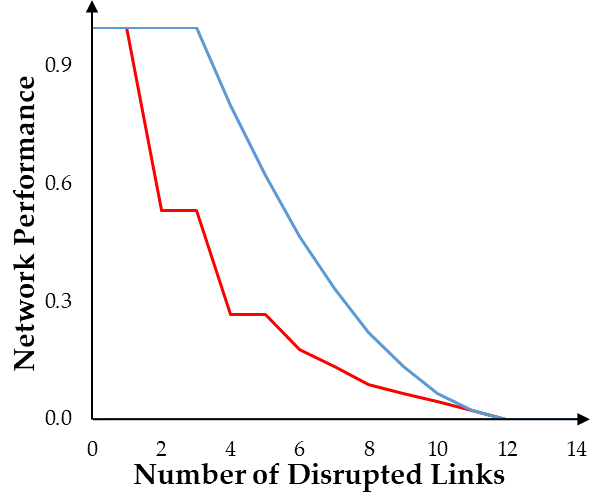 | 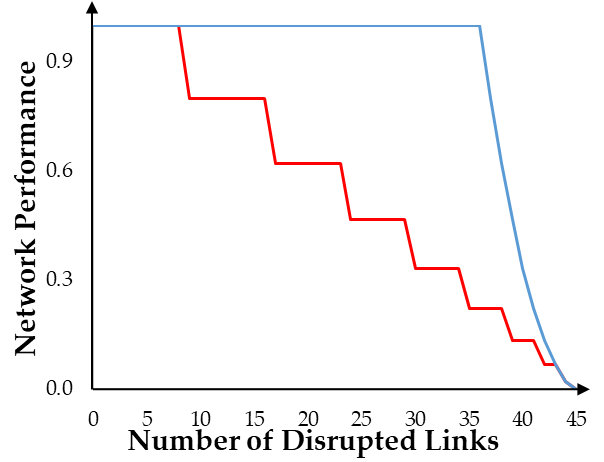 | 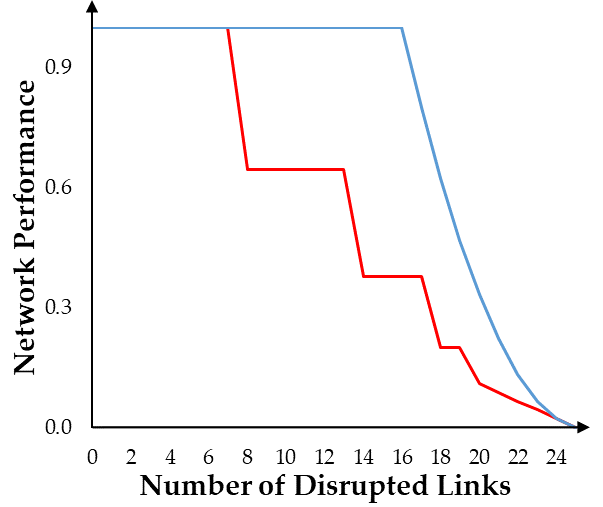 | 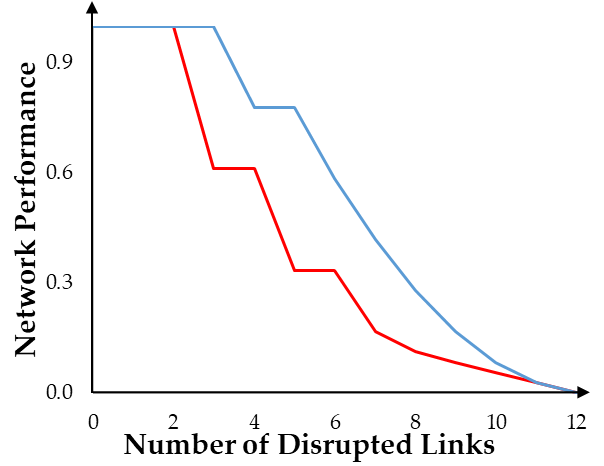 |
| **Complete Grid** | **Grid** | **Scale-Free** | **Random** |
| 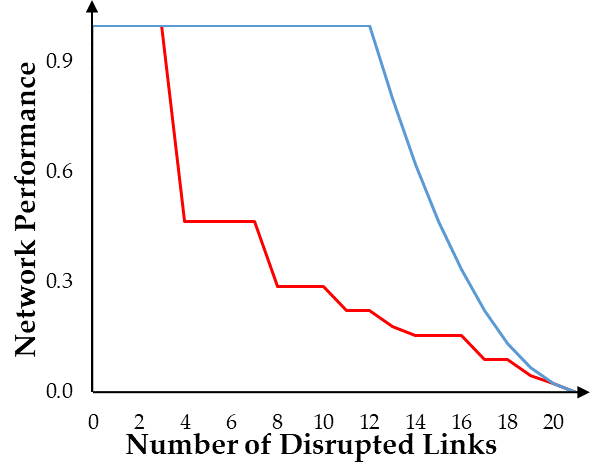 | 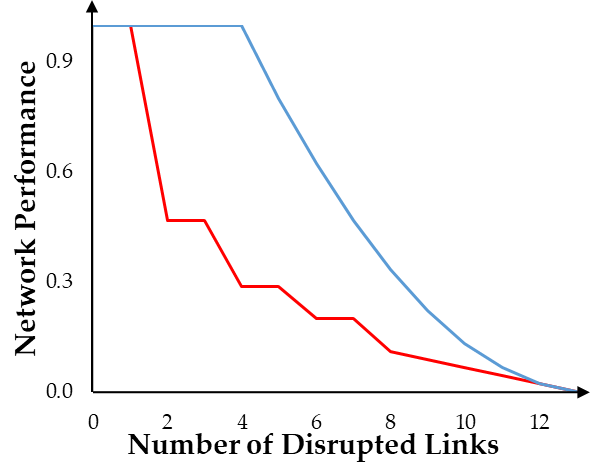 | 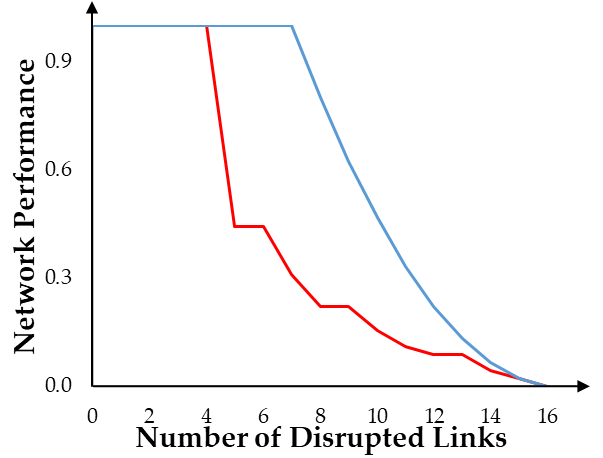 | 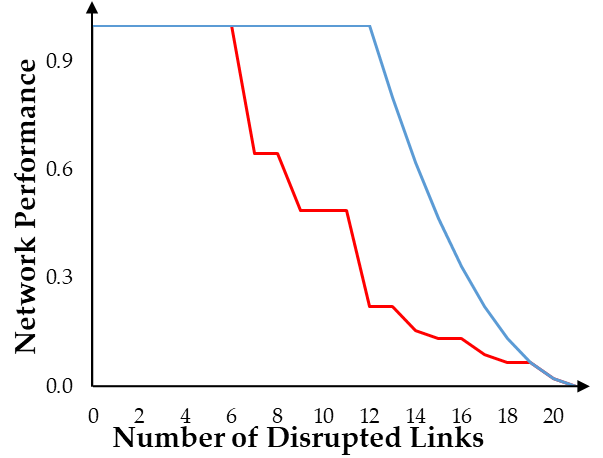 |
| **FIGURE 1** Disruption trajectory of 16 network topologies with 10 nodes. The x-axis represents the number of removed links. The y-axis represents the normalized network performance. | | | |

| **Diverging Tail** | **Tree** | **Crossing Path** | **Hub-and-Spoke** |
| --- | --- | --- | --- |
| 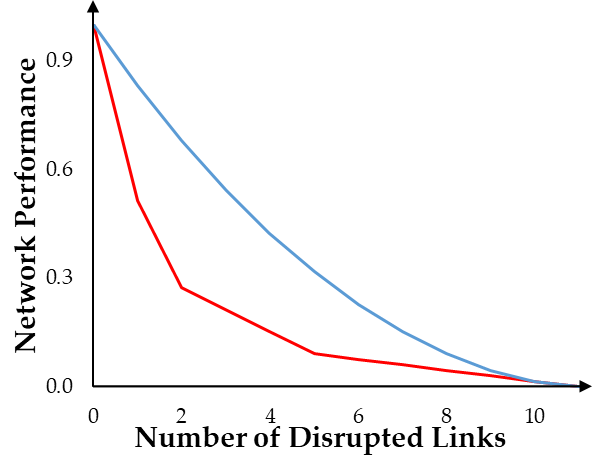 | 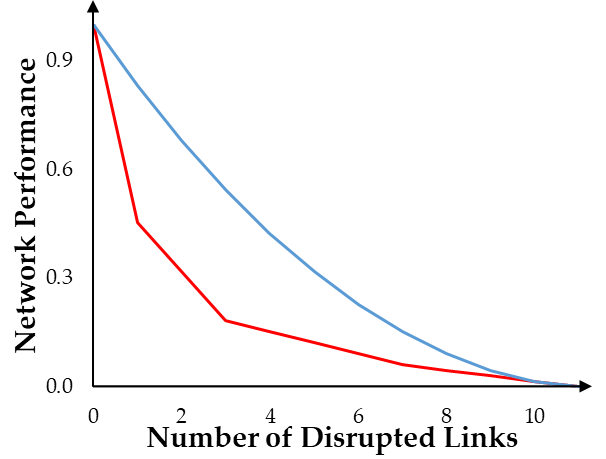 | 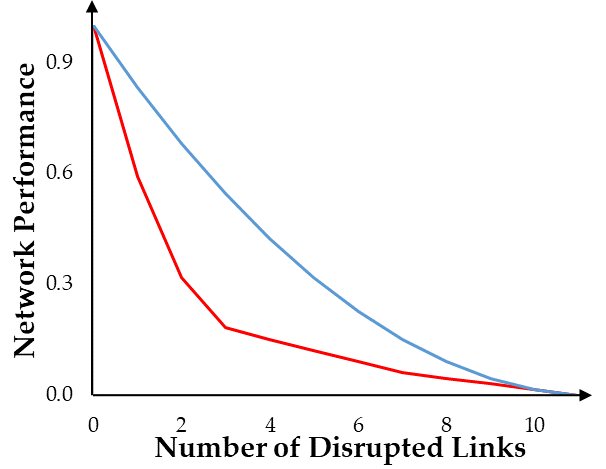 | 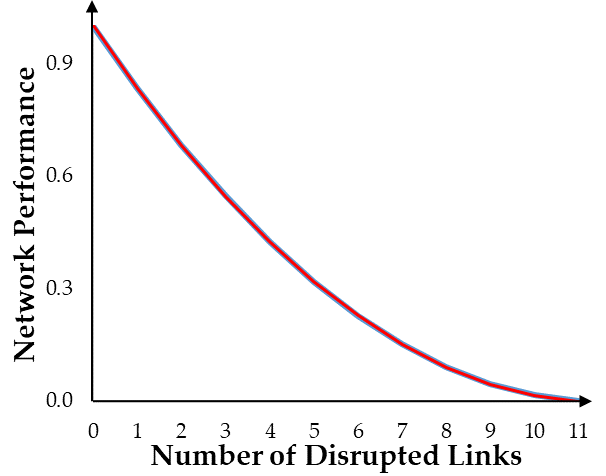 |
| **Central Ring** | **Converging Tail** | **Double U** | **Ring** |
| 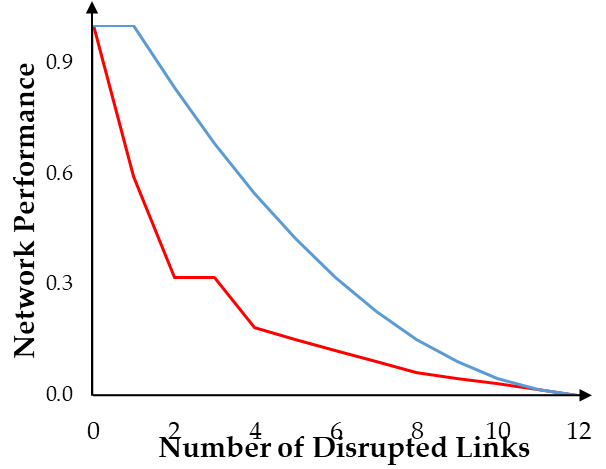 | 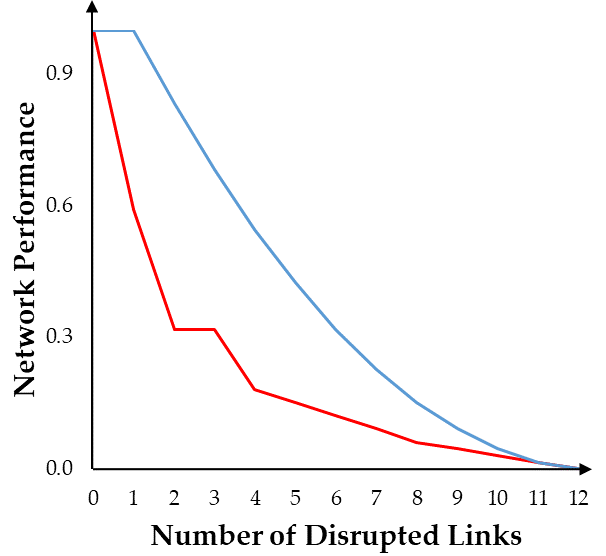 | 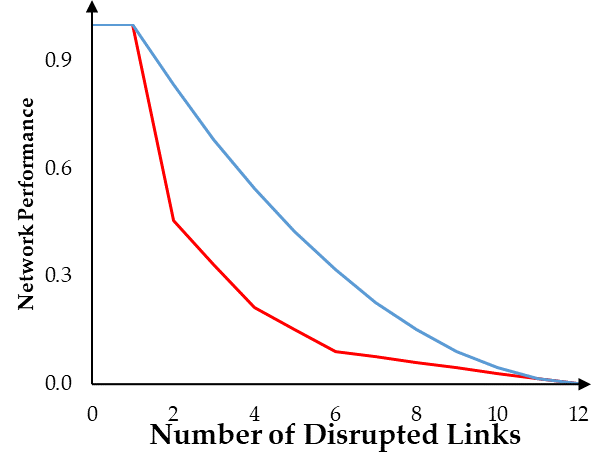 | 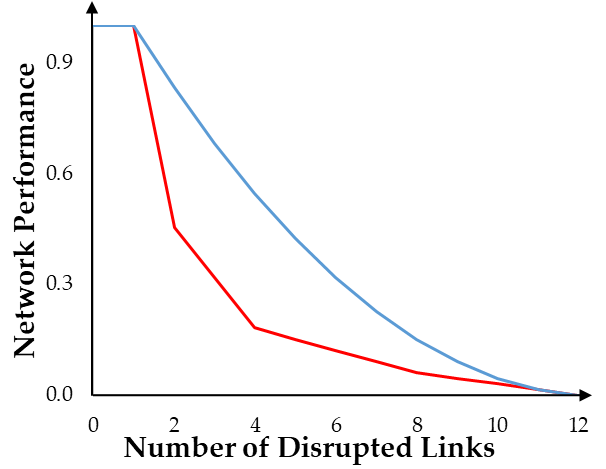 |
| **Single Depot** | **Complete** | **Matching Pairs** | **Diamond** |
| 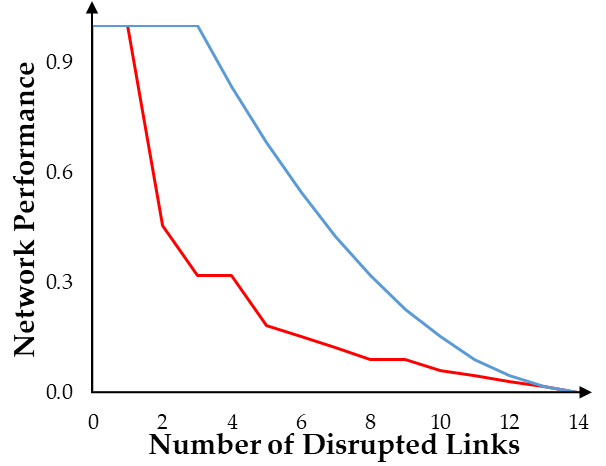 | 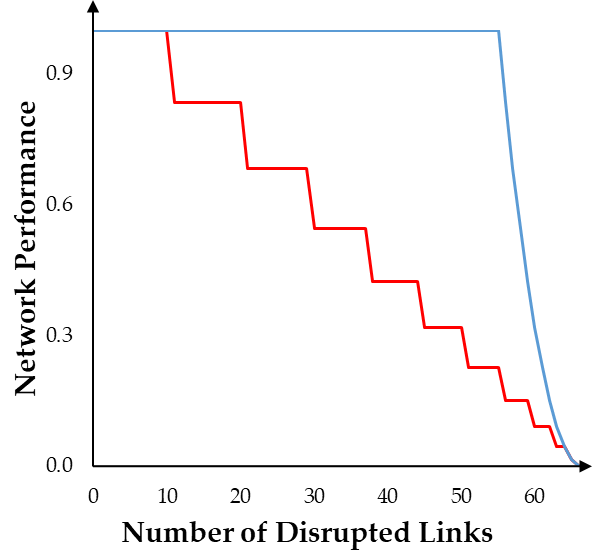 | 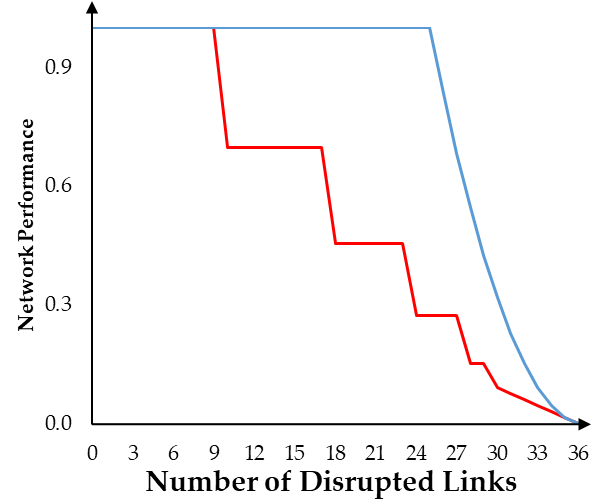 | 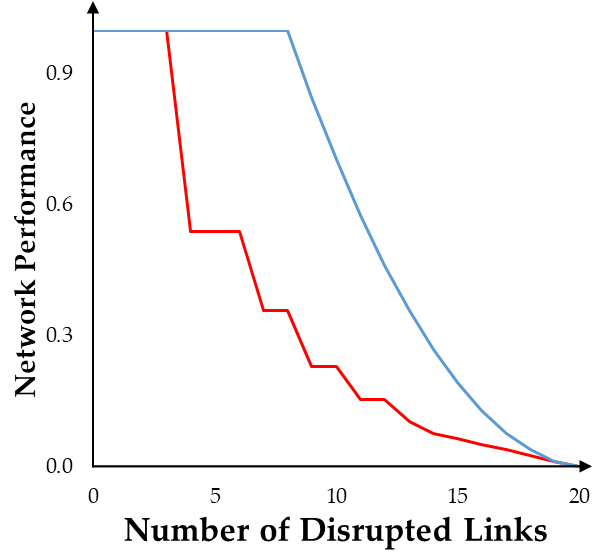 |
| **Complete Grid** | **Grid** | **Scale-Free** | **Random** |
| 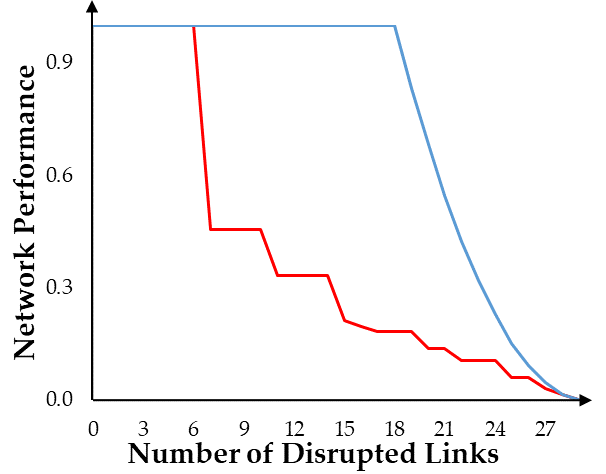 | 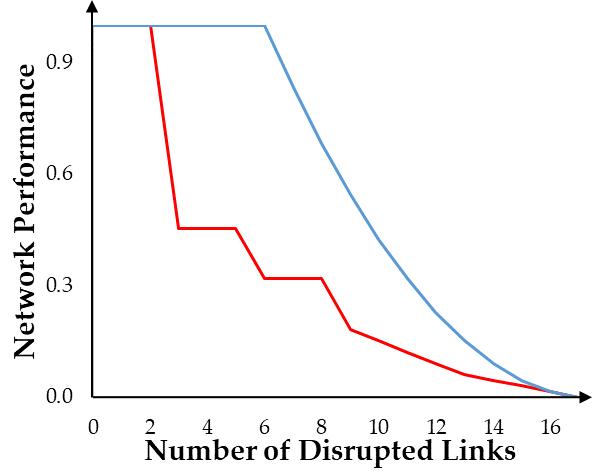 | 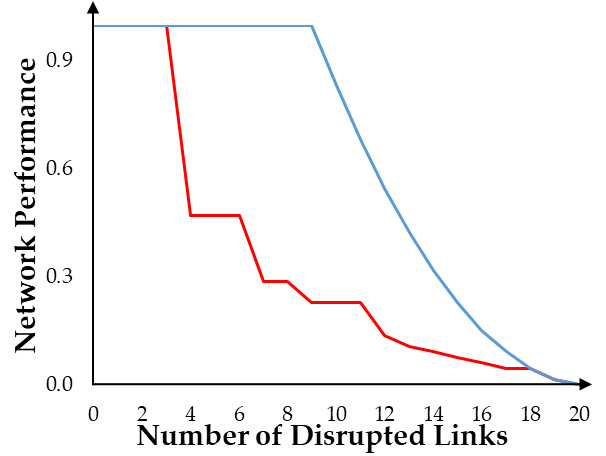 | 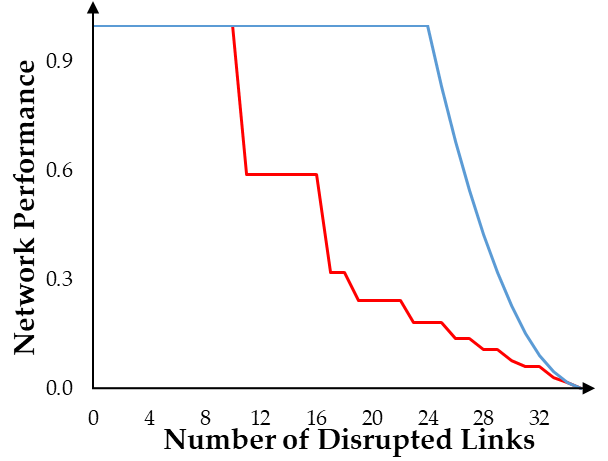 |
| **FIGURE 2** Disruption trajectory of 16 network topologies with 12 nodes. The x-axis represents the number of removed links. The y-axis represents the normalized network performance. | | | |

| **Diverging Tail** | **Tree** | **Crossing Path** | **Hub-and-Spoke** |
| --- | --- | --- | --- |
| 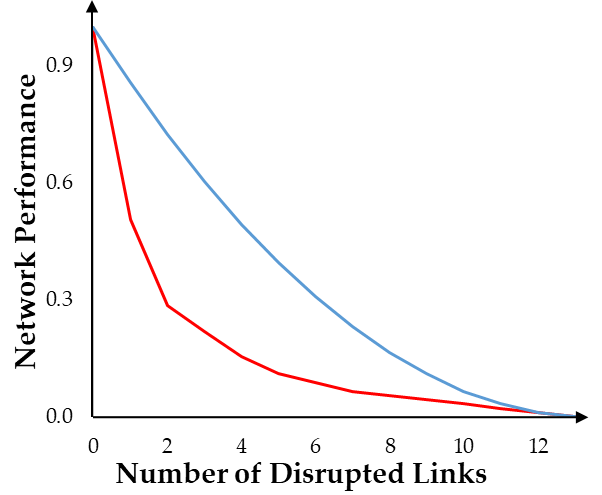 | 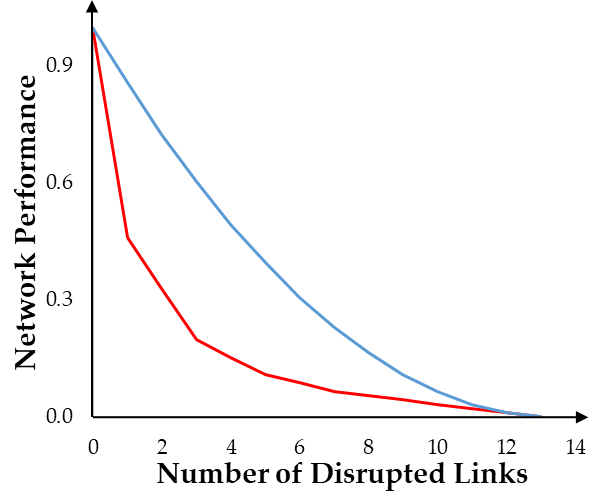 | 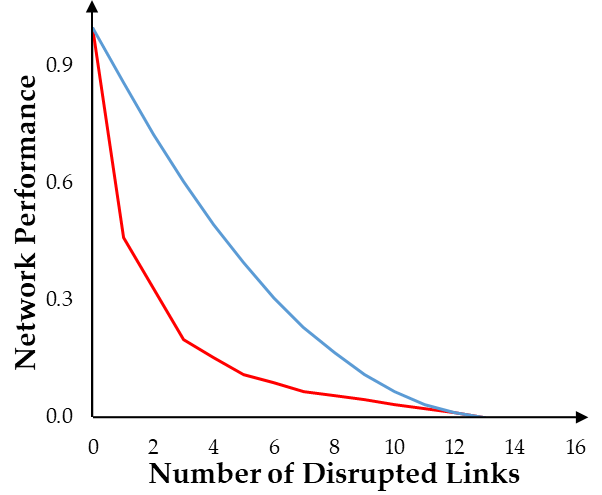 | 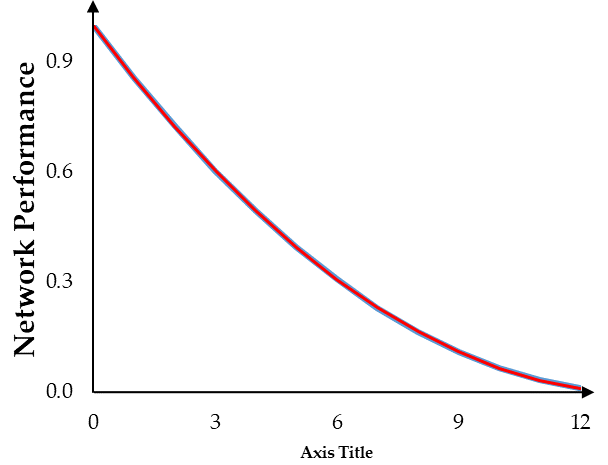 |
| **Central Ring** | **Converging Tail** | **Double U** | **Ring** |
| 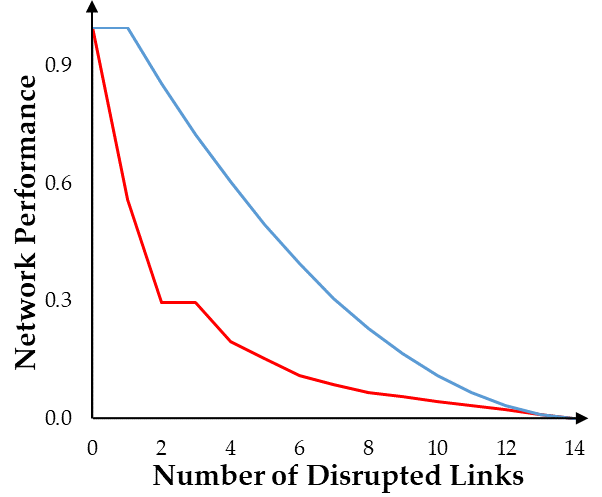 | 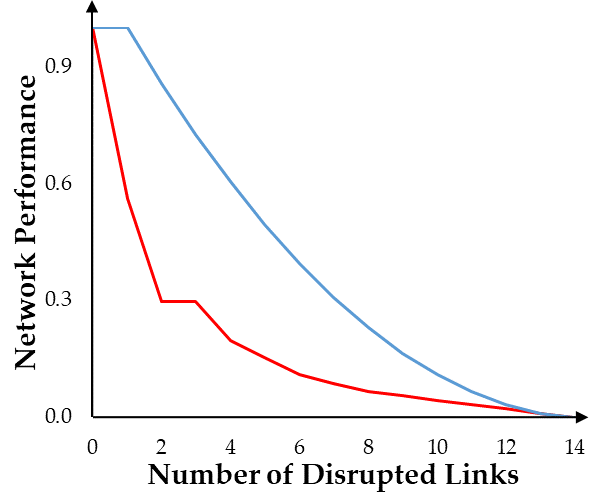 | 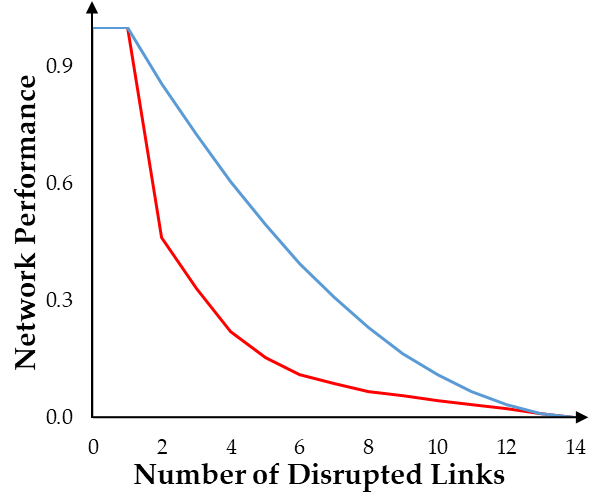 | 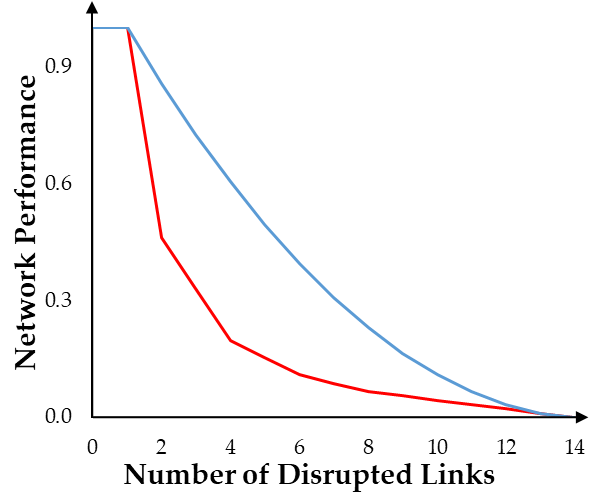 |
| **Single Depot** | **Complete** | **Matching Pairs** | **Diamond** |
| 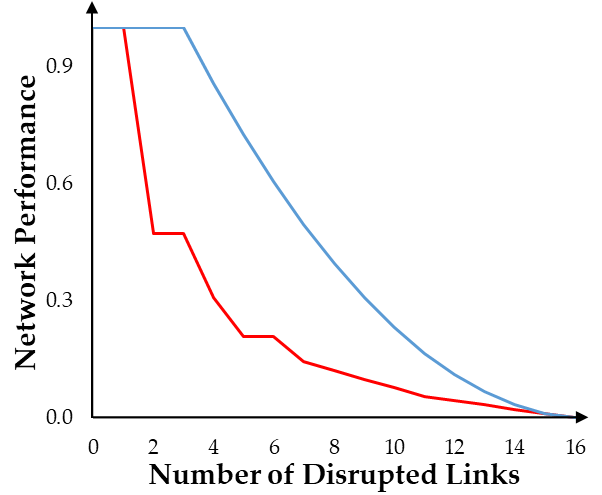 | 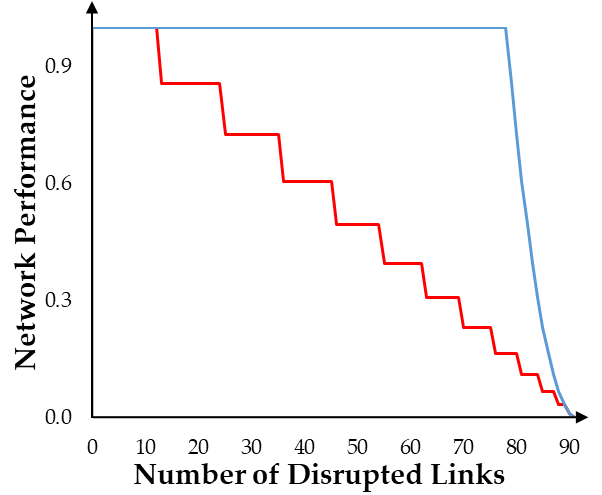 | 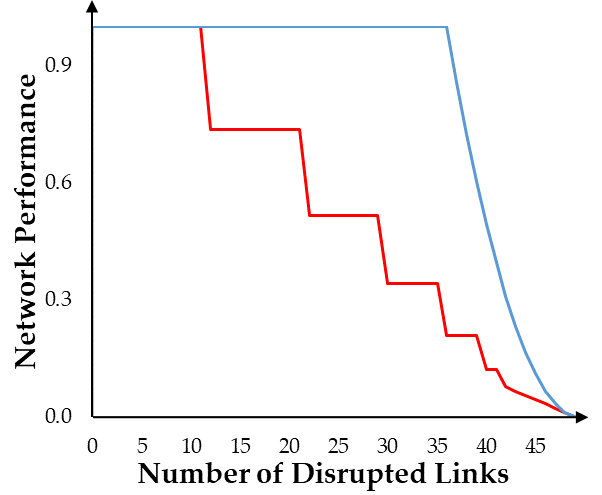 | 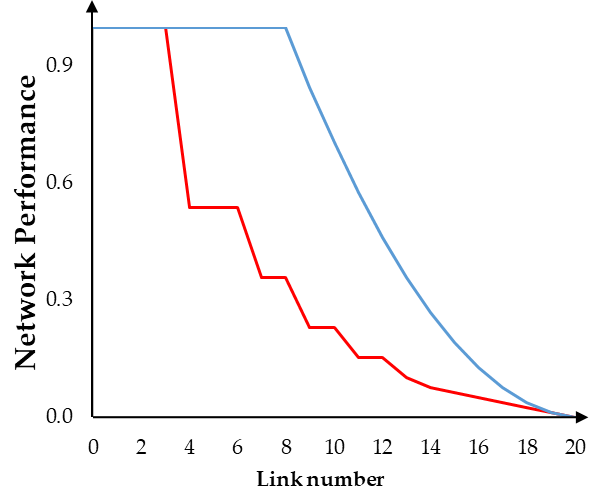 |
| **Complete Grid** | **Grid** | **Scale-Free** | **Random** |
| 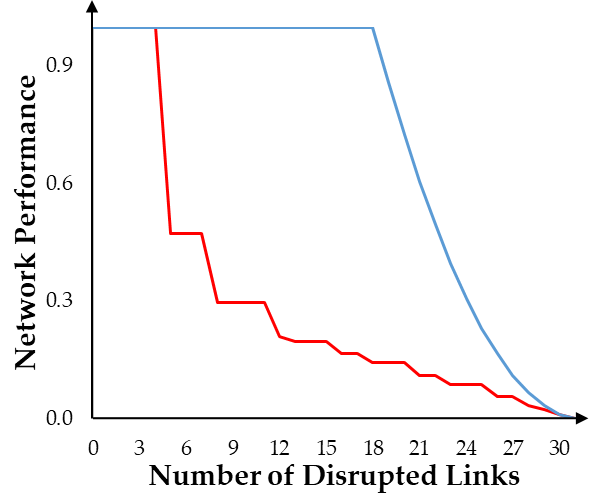 | 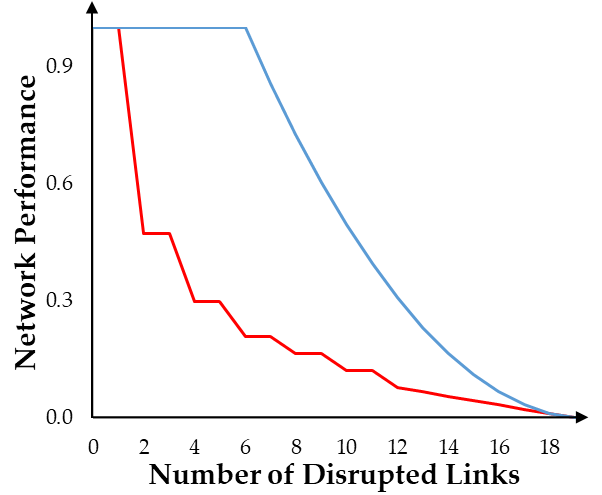 | 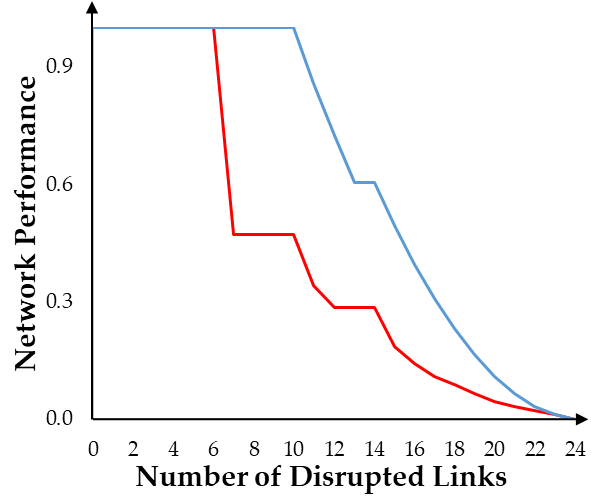 | 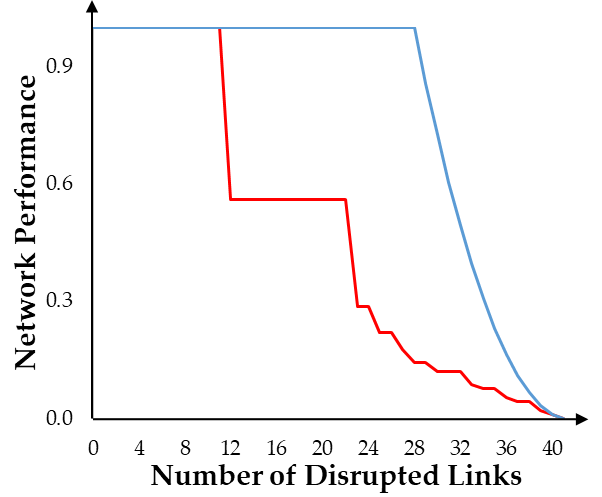 |
| **FIGURE 3** Disruption trajectory of 16 network topologies with 14 nodes. The x-axis represents the number of removed links. The y-axis represents the normalized network performance. | | | |

| **Diverging Tail** | **Tree** | **Crossing Path** | **Hub-and-Spoke** |
| --- | --- | --- | --- |
| 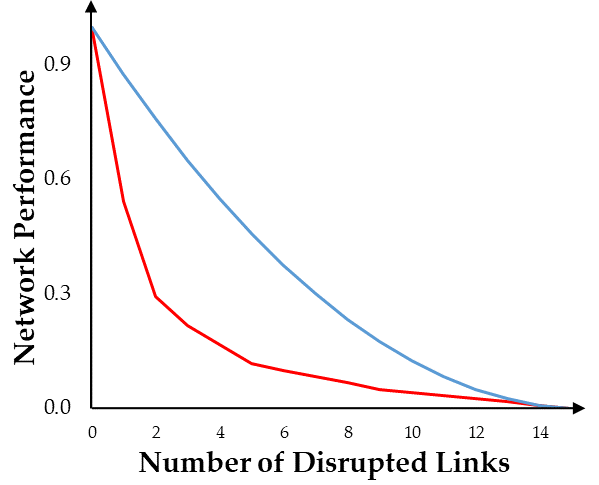 | 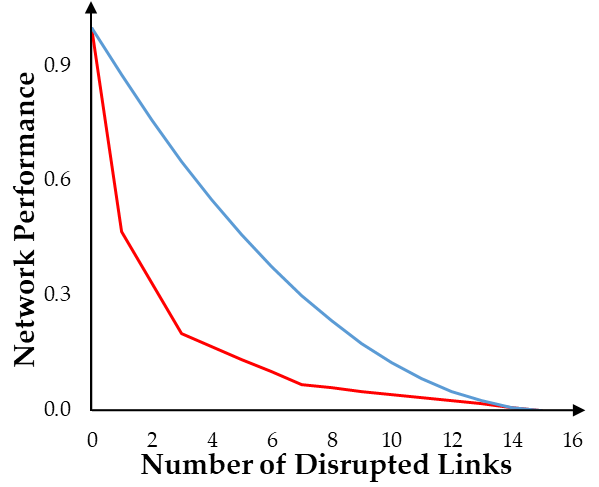 | 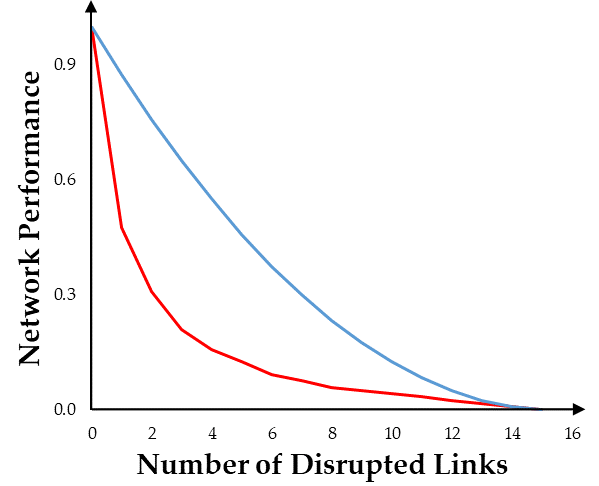 | 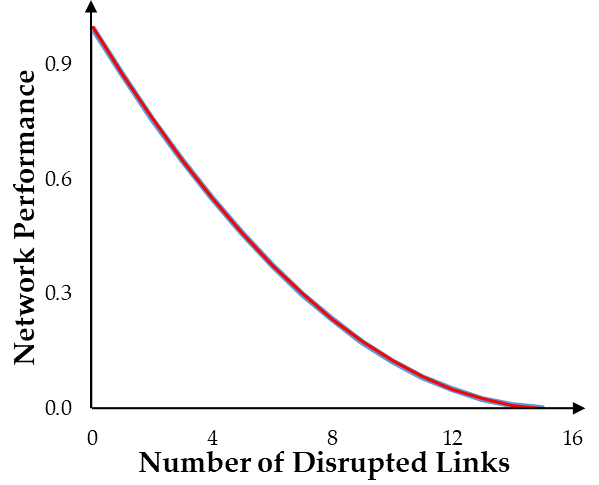 |
| **Central Ring** | **Converging Tail** | **Double U** | **Ring** |
| 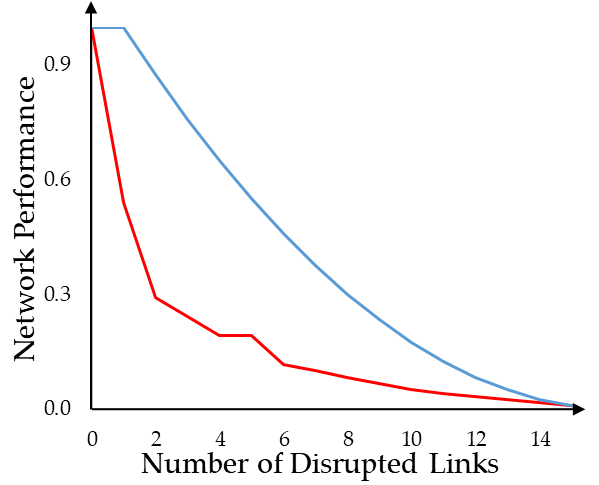 | 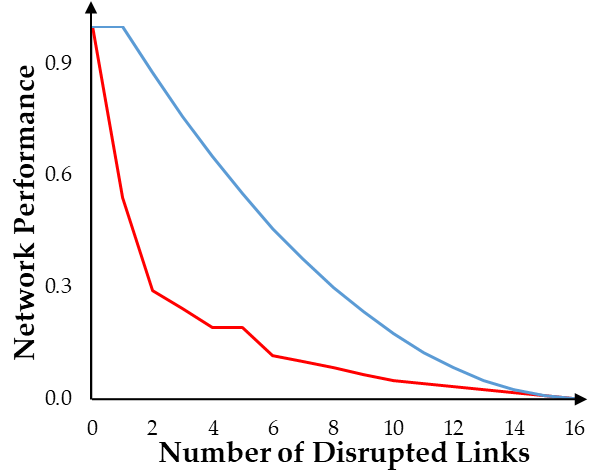 | 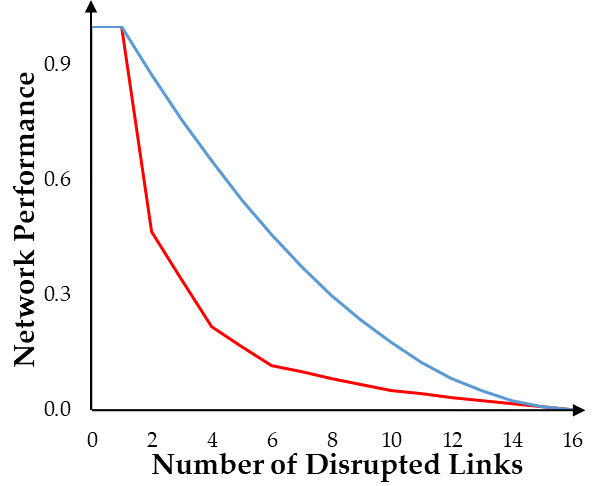 | 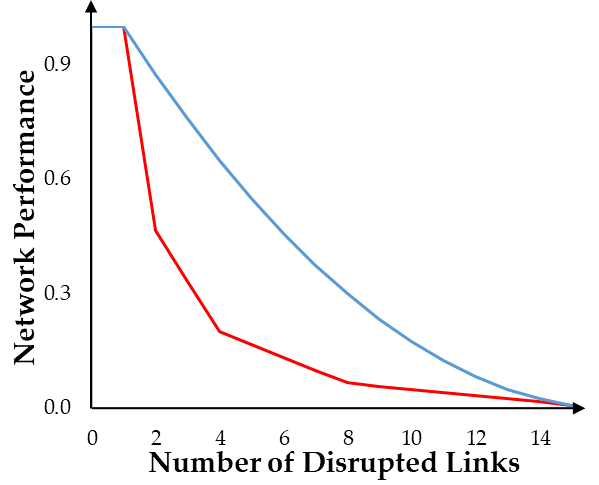 |
| **Single Depot** | **Complete** | **Matching Pairs** | **Diamond** |
| 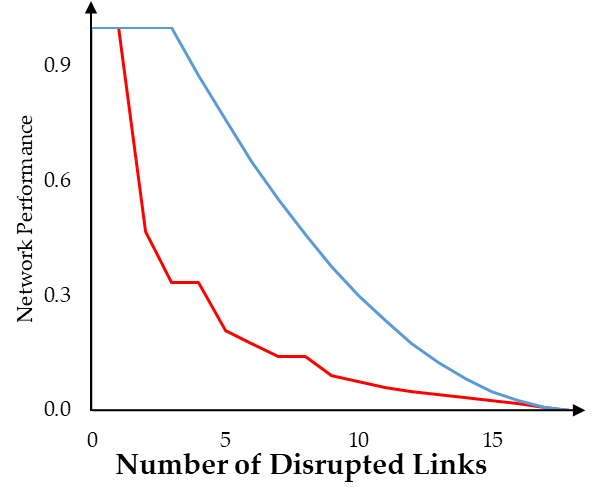 | 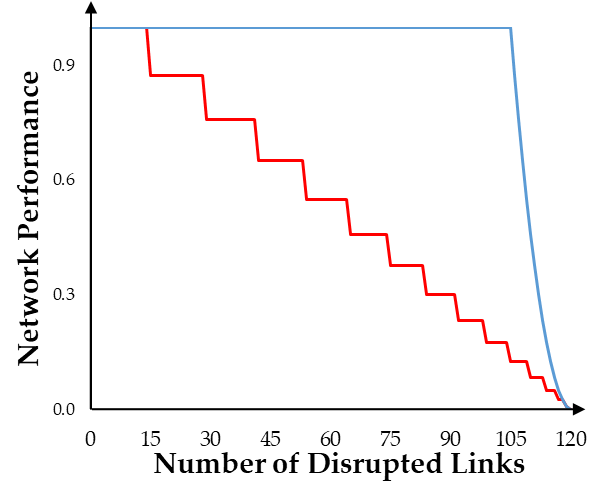 | 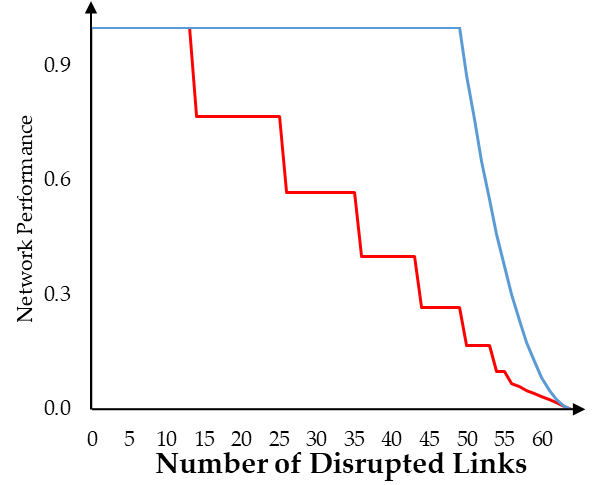 | 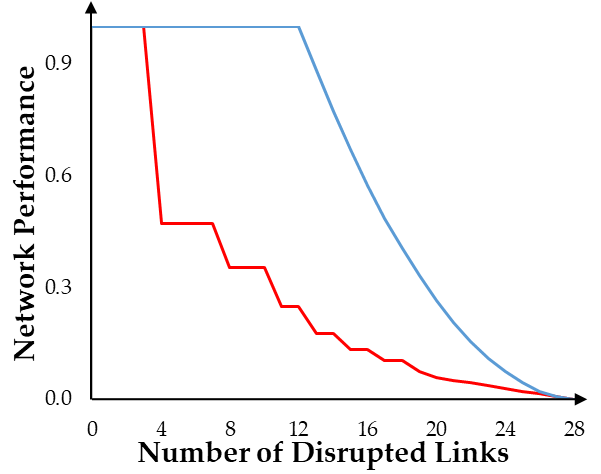 |
| **Complete Grid** | **Grid** | **Scale-Free** | **Random** |
| 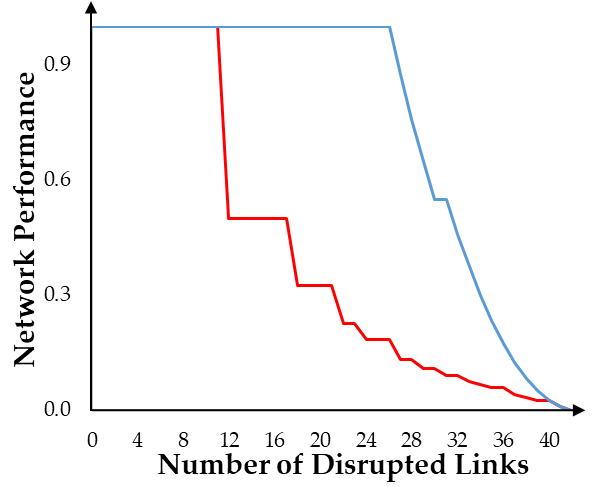 | 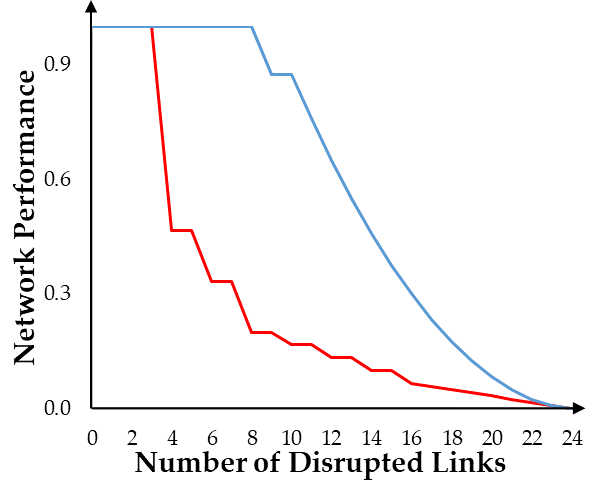 | 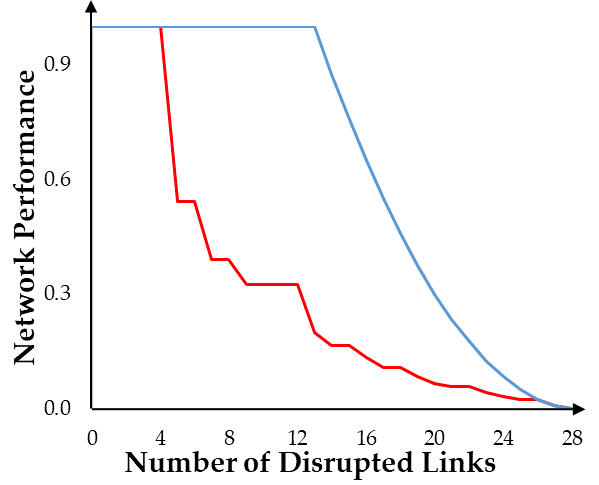 | 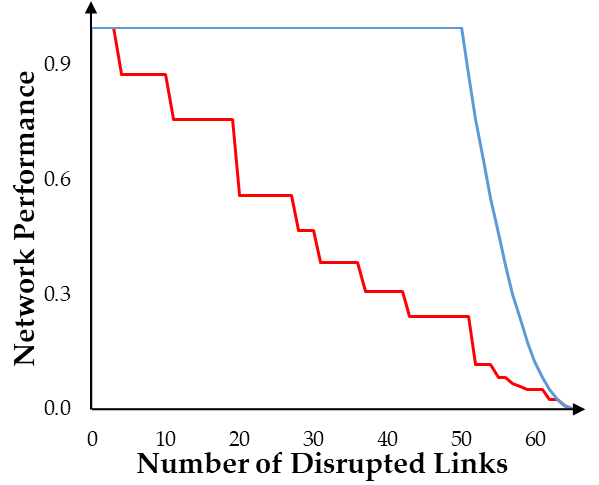 |
| **FIGURE 4** Disruption trajectory of 16 network topologies with 16 nodes. The x-axis represents the number of removed links. The y-axis represents the normalized network performance. | | | |

| **Diverging Tail** | **Tree** | **Crossing Path** | **Hub-and-Spoke** |
| --- | --- | --- | --- |
| 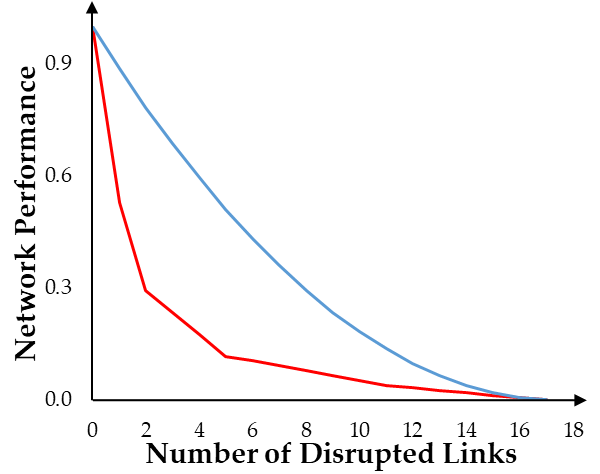 | 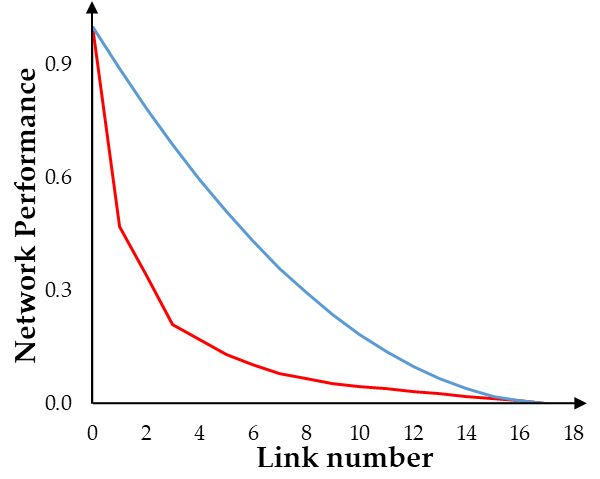 | 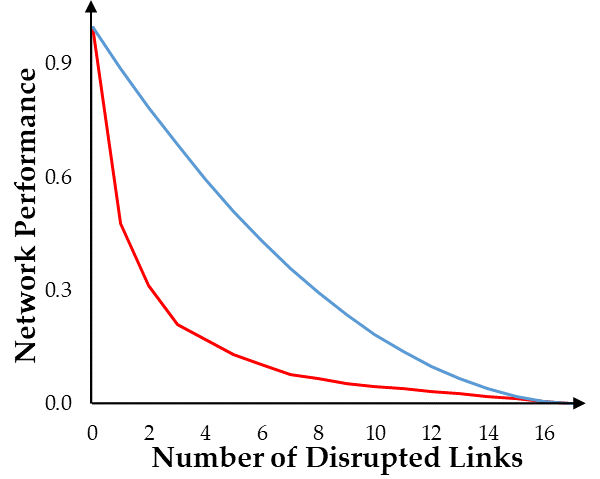 | 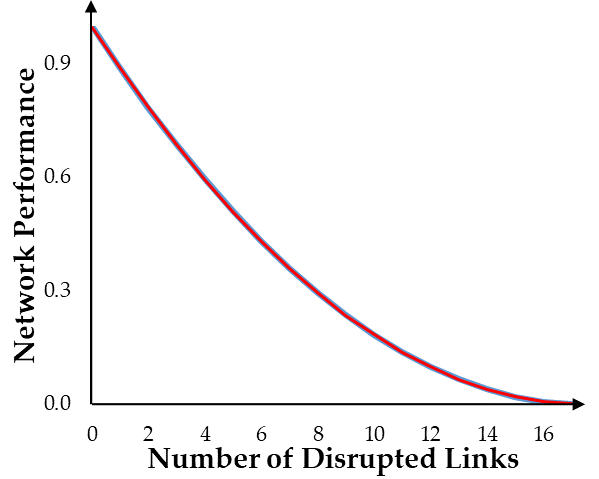 |
| **Central Ring** | **Converging Tail** | **Double U** | **Ring** |
| 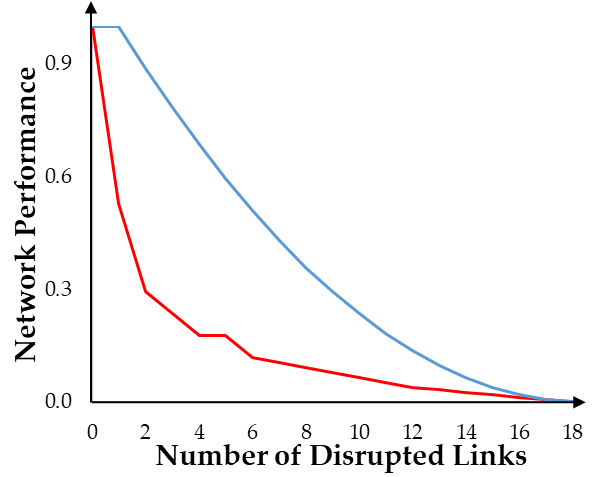 | 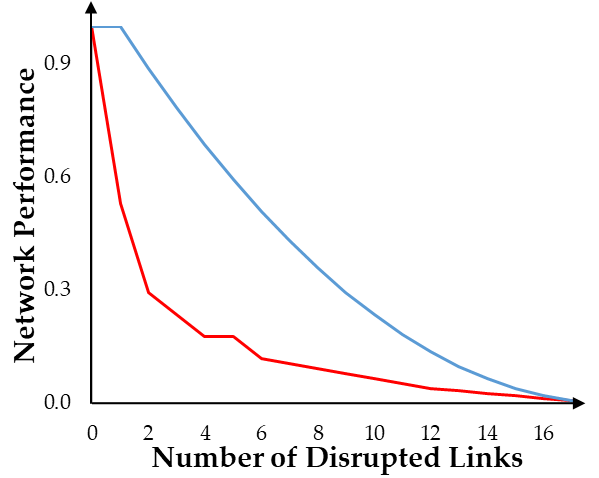 | 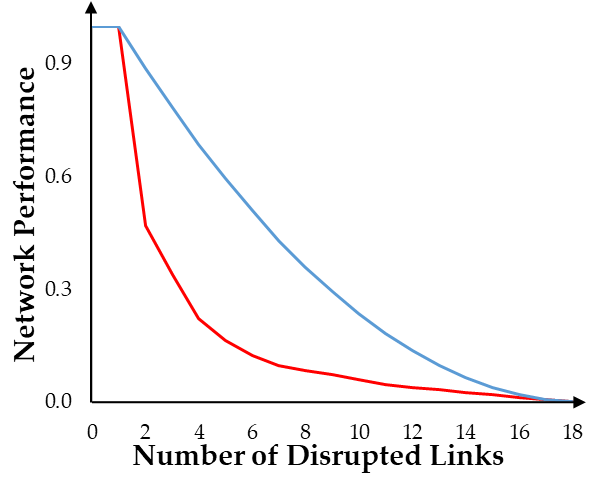 | 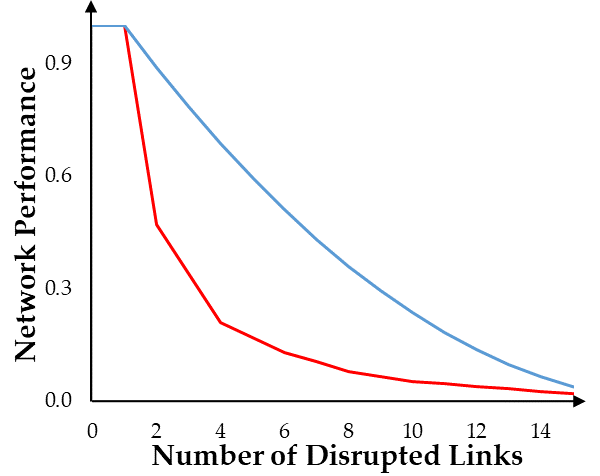 |
| **Single Depot** | **Complete** | **Matching Pairs** | **Diamond** |
| 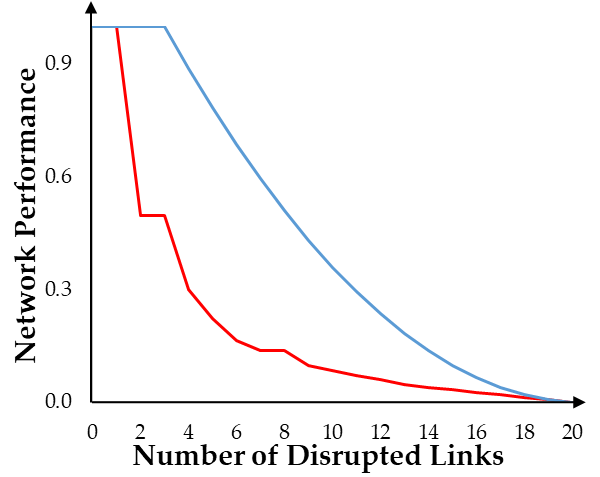 | 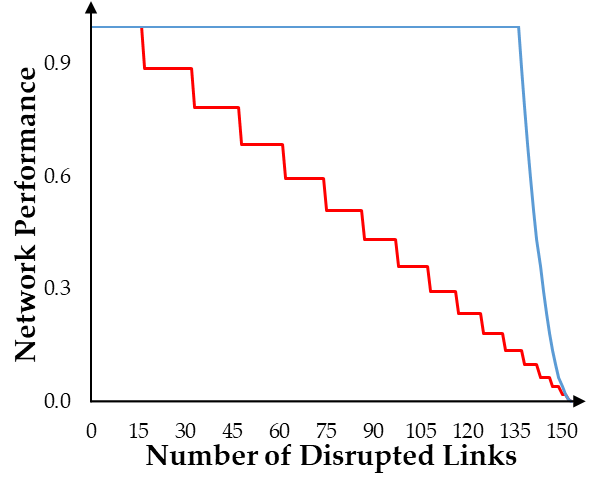 | 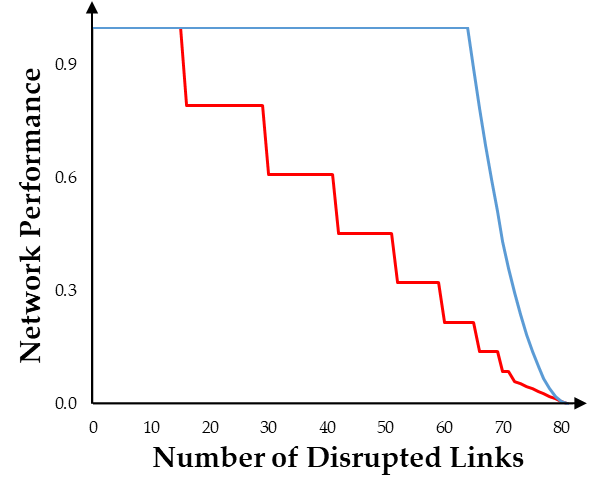 | 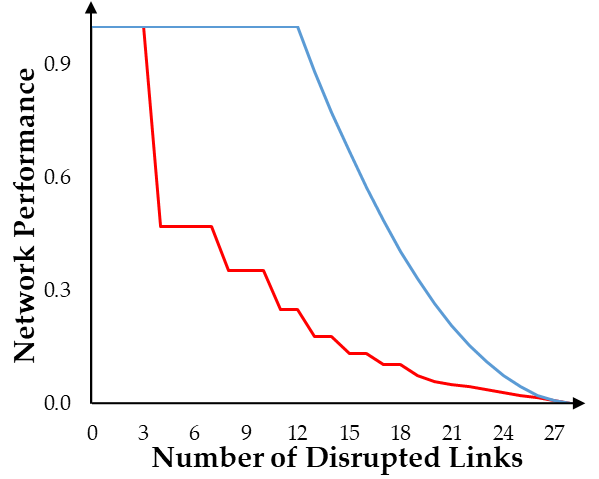 |
| **Complete Grid** | **Grid** | **Scale-Free** | **Random** |
| 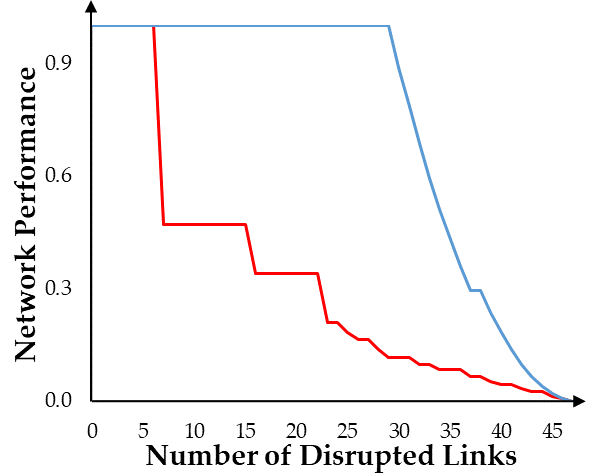 | 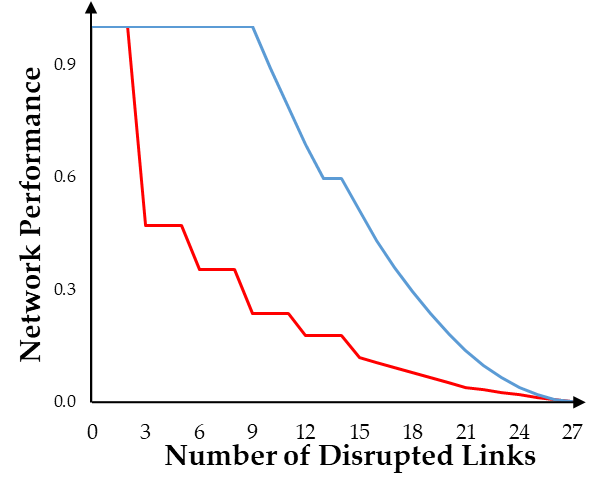 | 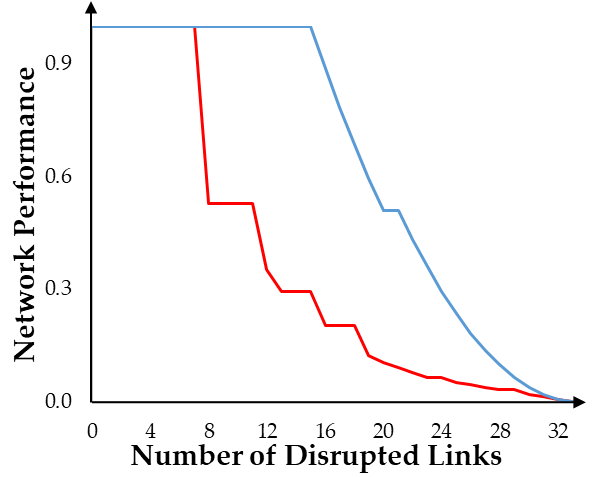 | 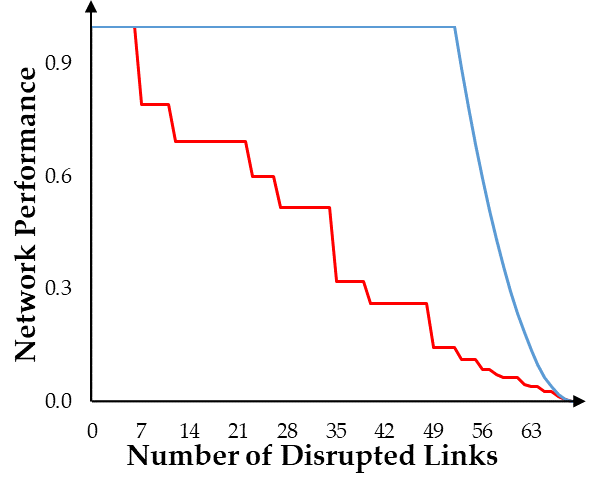 |
| **FIGURE 5** Disruption trajectory of 16 network topologies with 18 nodes. The x-axis represents the number of removed links. The y-axis represents the normalized network performance. | | | |

| **Diverging Tail** | **Tree** | **Crossing Path** | **Hub-and-Spoke** |
| --- | --- | --- | --- |
| 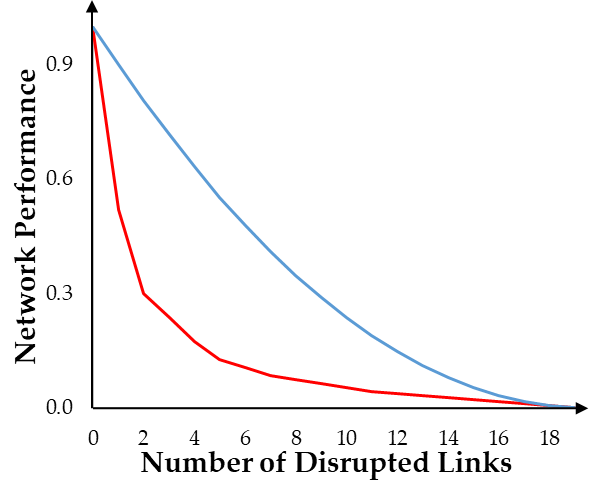 | 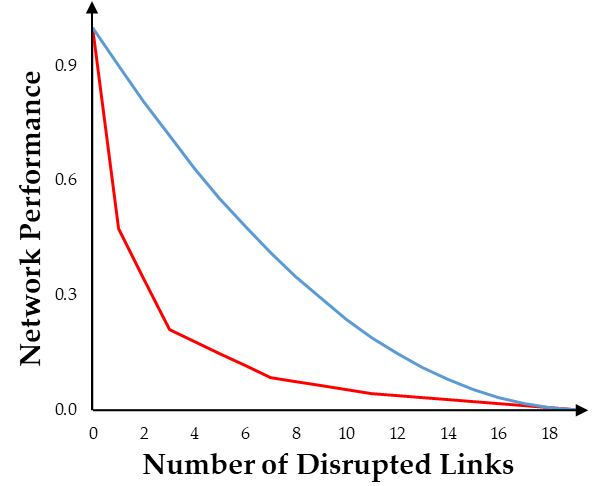 |  |  |
| **Central Ring** | **Converging Tail** | **Double U** | **Ring** |
|  |  |  |  |
| **Single Depot** | **Complete** | **Matching Pairs** | **Diamond** |
|  |  |  |  |
| **Complete Grid** | **Grid** | **Scale-Free** | **Random** |
|  |  |  |  |
| **FIGURE 6** Disruption trajectory of 16 network topologies with 20 nodes. The x-axis represents the number of removed links. The y-axis represents the normalized network performance. | | | |

| **Diverging Tail** | **Tree** | **Crossing Path** | **Hub-and-Spoke** |
| --- | --- | --- | --- |
|  |  |  |  |
| **Central Ring** | **Converging Tail** | **Double U** | **Ring** |
|  |  |  |  |
| **Single Depot** | **Complete** | **Matching Pairs** | **Diamond** |
|  |  |  |  |
| **Complete Grid** | **Grid** | **Scale-Free** | **Random** |
|  |  |  |  |
| **FIGURE 7** Disruption trajectory of 16 network topologies with 22 nodes. The x-axis represents the number of removed links. The y-axis represents the normalized network performance. | | | |

| **Diverging Tail** | **Tree** | **Crossing Path** | **Hub-and-Spoke** |
| --- | --- | --- | --- |
|  |  |  |  |
| **Central Ring** | **Converging Tail** | **Double U** | **Ring** |
|  |  |  |  |
| **Single Depot** | **Complete** | **Matching Pairs** | **Diamond** |
|  |  |  |  |
| **Complete Grid** | **Grid** | **Scale-Free** | **Random** |
|  |  |  |  |
| **FIGURE 8** Disruption trajectory of 16 network topologies with 24 nodes. The x-axis represents the number of removed links. The y-axis represents the normalized network performance. | | | |

| **Diverging Tail** | **Tree** | **Crossing Path** | **Hub-and-Spoke** |
| --- | --- | --- | --- |
|  |  |  |  |
| **Central Ring** | **Converging Tail** | **Double U** | **Ring** |
|  |  |  |  |
| **Single Depot** | **Complete** | **Matching Pairs** | **Diamond** |
|  |  |  |  |
| **Complete Grid** | **Grid** | **Scale-Free** | **Random** |
|  |  |  |  |
| **FIGURE 9** Disruption trajectory of 16 network topologies with 26 nodes. The x-axis represents the number of removed links. The y-axis represents the normalized network performance. | | | |

**Supplementary Information IV**

Vulnerability under benign attack and uncertainty in vulnerability bivariate analysis.

|  |  |
| --- | --- |
| N=8 | N=10 |
|  |  |
| N=12 | N=14 |
|  |  |
| N=16 | N=18 |
|  |  |
| N=20 | N=22 |
|  |  |
| N=24 | N=26 |
| **FIGURE 1** Bivariate analysis between vulnerability under benign attack and uncertainty in vulnerability measures for different topologies and network sizes. | |
